# Effect of statin therapy on muscle symptoms: an individual participant data meta-analysis of large-scale, randomised, double-blind trials

**DOI:** 10.1016/S0140-6736(22)01545-8

**Published:** 2022-09

**Authors:** Cholesterol Treatment Trialists’ Collaboration, Writing committee:, Christina Reith, Colin Baigent, Lisa Blackwell, Jonathan Emberson, Enti Spata, Kelly Davies, Heather Halls, Lisa Holland, Kate Wilson, Jane Armitage, Charlie Harper, David Preiss, Alistair Roddick, Anthony Keech, John Simes, Rory Collins, CTT secretariat:, Jane Armitage, Colin Baigent, Elizabeth Barnes, Lisa Blackwell, Rory Collins, Kelly Davies, Jonathan Emberson, Jordan Fulcher, Heather Halls, Charlie Harper, William G Herrington, Lisa Holland, Anthony Keech, Adrienne Kirby, Borislava Mihaylova, Rachel O’Connell, David Preiss, Christina Reith, John Simes, Enti Spata, Kate Wilson, Independent oversight committee:, Emily Banks, Michael Blastland, Stephen Evans, Robert Temple, Peter Weissberg, Janet Wittes, Collaborating trials: A to Z trial (phase Z), Michael Blazing, Eugene Braunwald, James de Lemos, Sabina Murphy, Terje R Pedersen, Marc Pfeffer, Harvey White, Stephen Wiviott, AFCAPS/TEXCAPS (AirForce/Texas Coronary Atherosclerosis Prevention Study), Michael Clearfield, John R Downs, Antonio Gotto Jr, Stephen Weis, ALERT (Assessment of Lescol in Renal Transplantation), Bengt Fellström, Hallvard Holdaas, Alan Jardine, Terje R Pedersen, ALLHAT (Antihypertensive and Lipid-Lowering Treatment to Prevent Heart Attack Trial), David Gordon, Barry Davis, Curt Furberg, Richard Grimm, Sara Pressel, Jeffrey Probstfield, Mahboob Rahman, Lara Simpson, ALLIANCE (Aggressive Lipid-Lowering Initiation Abates New Cardiac Events), Michael Koren, ASCOT (Anglo-Scandinavian Cardiac Outcomes Trial), Björn Dahlöf, Ajay Gupta, Neil Poulter, Peter Sever, Hans Wedel, ASPEN (Atorvastatin Study for the Prevention of Coronary Heart Disease Endpoints in Non-Insulin Dependent Diabetes Mellitus), Robert H Knopp, AURORA (A study to evaluate the Use of Rosuvastatin in subjects On Regular haemodialysis: an Assessment of survival and cardiovascular events), Stuart Cobb, Bengt Fellström, Hallvard Holdaas, Alan Jardine, Roland Schmieder, Faiez Zannad, CARDS (Collaborative Atorvastatin Diabetes Study), D John Betteridge, Helen M Colhoun, Paul N Durrington, John Fuller, Graham A Hitman, Andrew Neil, CARE (Cholesterol And Recurrent Events Study), Eugene Braunwald, Barry Davis, C Morton Hawkins, Lemuel Moyé, Marc Pfeffer, Frank Sacks, CORONA (Controlled Rosuvastatin Multinational Trial in Heart Failure), John Kjekshus, Hans Wedel, John Wikstrand, 4D (Die Deutsche Diabetes Dialyse Studie), Christoph Wanner, Vera Krane, GISSI (Gruppo Italiano per lo Studio della Sopravvivenza nell’Infarto miocardico) Heart Failure and Prevention trials, Maria Grazia Franzosi, Roberto Latini, Donata Lucci, Aldo Maggioni, Roberto Marchioli, Enrico B Nicolis, Luigi Tavazzi, Gianni Tognoni, HOPE-3, Jackie Bosch, Eva Lonn, Salim Yusuf, HPS (Heart Protection Study), Jane Armitage, Louise Bowman, Rory Collins, Anthony Keech, Martin Landray, Sarah Parish, Richard Peto, Peter Sleight, IDEAL (Incremental Decrease in Endpoints through Aggressive Lipid-lowering), John JP Kastelein, Terje R Pedersen, JUPITER (Justificationfor the Use of Statins in Prevention: an Intervention Trial Evaluating Rosuvastatin), Robert Glynn, Antonio Gotto Jr, John JP Kastelein, Wolfgang Koenig, Jean Macfadyen, Paul M Ridker, LIPID (Long-term Intervention with Pravastatin in Ischaemic Disease), Anthony Keech, Stephen MacMahon, Ian Marschner, John Shaw, John Simes, Andrew Tonkin, Harvey White, LIPS (Lescol Intervention Prevention Study), Patrick W Serruys, Post-CABG (Post-Coronary Artery Bypass Graft Study), Genell Knatterud, PROSPER (Prospective Study of Pravastatin in the Elderly at Risk), Naveed Sataar, J Wouter Jukema, Stella Trompet, Ian Ford, PROVE-IT (Pravastatin or Atorvastatin Evaluation and Infection Therapy), Eugene Braunwald, Christopher P Cannon, Sabina Murphy, SEARCH (Study of Effectiveness of Additional Reductions in Cholesterol and Homocysteine), Rory Collins, Jane Armitage, Louise Bowman, Sarah Parish, Richard Peto, Peter Sleight, SPARCL (Stroke Prevention by Aggressive Reduction in Cholesterol Levels), Pierre Amarenco, K Michael Welch, 4S Scandinavian Simvastatin Survival Study), John Kjekshus, Terje R Pedersen, Lars Wilhelmsen, TNT (Treating to New Targets), Phlip Barter, Antonio Gotto JR, John La Rosa, John JP Kastelein, James Shepherd, WOSCOPS (West of Scotland Coronary Prevention Study), Stuart Cobbe, Ian Ford, Sharon Kean, Peter Macfarlane, Chris Packard, Michele Roberston, Naveed Sattar, James Shepherd, Robin Young, Other CTT Members:, Hiroyuki Arashi, Robert Byington, Robert Clarke, Marcus Flather, Shinya Goto, Uri Goldbourt, Jemma Hopewell, Kees Hovingh, Patricia Kearney, George Kitas, Connie Newman, Marc S Sabatine, Greg Schwartz, Liam Smeeth, Jonathan Tobert, John Varigos, Junichi Yamaguchi

## Abstract

**Background:**

Statin therapy is effective for the prevention of atherosclerotic cardiovascular disease and is widely prescribed, but there are persisting concerns that statin therapy might frequently cause muscle pain or weakness. We aimed to address these through an individual participant data meta-analysis of all recorded adverse muscle events in large, long-term, randomised, double-blind trials of statin therapy.

**Methods:**

Randomised trials of statin therapy were eligible if they aimed to recruit at least 1000 participants with a scheduled treatment duration of at least 2 years, and involved a double-blind comparison of statin versus placebo or of a more intensive versus a less intensive statin regimen. We analysed individual participant data from 19 double-blind trials of statin versus placebo (n=123 940) and four double-blind trials of a more intensive versus a less intensive statin regimen (n=30 724). Standard inverse-variance-weighted meta-analyses of the effects on muscle outcomes were conducted according to a prespecified protocol.

**Findings:**

Among 19 placebo-controlled trials (mean age 63 years [SD 8], with 34 533 [27·9%] women, 59 610 [48·1%] participants with previous vascular disease, and 22 925 [18·5%] participants with diabetes), during a weighted average median follow-up of 4·3 years, 16 835 (27·1%) allocated statin versus 16 446 (26·6%) allocated placebo reported muscle pain or weakness (rate ratio [RR] 1·03; 95% CI 1·01–1·06). During year 1, statin therapy produced a 7% relative increase in muscle pain or weakness (1·07; 1·04–1·10), corresponding to an absolute excess rate of 11 (6–16) events per 1000 person-years, which indicates that only one in 15 ([1·07–1·00]/1·07) of these muscle-related reports by participants allocated to statin therapy were actually due to the statin. After year 1, there was no significant excess in first reports of muscle pain or weakness (0·99; 0·96–1·02). For all years combined, more intensive statin regimens (ie, 40–80 mg atorvastatin or 20–40 mg rosuvastatin once per day) yielded a higher RR than less intensive or moderate-intensity regimens (1·08 [1·04–1·13] *vs* 1·03 [1·00–1·05]) compared with placebo, and a small excess was present (1·05 [0·99–1·12]) for more intensive regimens after year 1. There was no clear evidence that the RR differed for different statins, or in different clinical circumstances. Statin therapy yielded a small, clinically insignificant increase in median creatine kinase values of approximately 0·02 times the upper limit of normal.

**Interpretation:**

Statin therapy caused a small excess of mostly mild muscle pain. Most (>90%) of all reports of muscle symptoms by participants allocated statin therapy were not due to the statin. The small risks of muscle symptoms are much lower than the known cardiovascular benefits. There is a need to review the clinical management of muscle symptoms in patients taking a statin.

**Funding:**

British Heart Foundation, Medical Research Council, Australian National Health and Medical Research Council.

## Introduction

Atherosclerotic cardiovascular diseases, chiefly myocardial infarction and ischaemic stroke, accounted for approximately 18 million deaths worldwide in 2019,^1^ and low-density lipoprotein (LDL) cholesterol is a major causal risk factor.^2^ Randomised controlled trials have shown that the long-term reduction of LDL cholesterol concentrations with an 3-hydroxy 3-methylglutaryl-coenzyme A reductase inhibitor (ie, a statin) reduces the incidence of myocardial infarction and of ischaemic stroke by approximately a quarter for every 1 mmol/L LDL cholesterol reduction achieved, which corresponds to the avoidance of approximately 50 major vascular events in those with pre-existing vascular disease (secondary prevention), and 25 major vascular events when used for primary prevention, in every 1000 people administered this therapy for 5 years.^2^ Moreover, a more intensive statin regimen (ie, 40–80 mg atorvastatin once per day, or 20–40 mg rosuvastatin once per day), which could reduce LDL cholesterol by 2 mmol/L, would prevent twice as many major vascular events, and longer treatment yields greater benefits. For a given reduction in LDL cholesterol, similar proportional reductions in risk are seen across a wide range of patients (including men and women, older and younger patients, and those with and without a previous history of cardiovascular disease).^3–6^ Consequently, statins are now used by millions of people worldwide.

It is known that statins can, rarely, cause substantial muscle damage (ie, myopathy [approximately one extra case per 10 000 person-years]; or, in a more severe form, rhabdomyolysis [approximately 2–3 cases per 100 000 person-years]) as indicated by muscle symptoms accompanied by biochemical changes (eg, multi-fold rises in creatine kinase).^2^ However, there are concerns regarding statin-related muscle adverse effects,^7^ although reviews of the data from randomised trials (including N-of-1 trials) have shown that most such muscle symptoms are due to the so-called nocebo or drucebo^8^effect (that is, that they are not generally due to the statin).^2,9^ Moreover, on the basis of non-randomised observational studies of routine health-care records, it has been suggested that statin therapy is associated with large excess risks of musculoskeletal disorders;^10–12^ and, although such studies are susceptible to statistical bias and confounding,^2^ they are often cited without reference to their limitations. In addition, most studies of statin intolerance, or of statin-associated muscle symptoms, report the proportions of patients who do not adhere to the statin regimen because of symptoms they attribute to a statin. However, such estimates have the potential to be misleading, because a proportion of such symptoms will not truly be due to the statin. Perhaps as a consequence, there is widespread disinformation and confusion among patients about statin safety.^13^

To provide more reliable information about the size, severity, and timing of any adverse effects caused by various statin regimens, the Cholesterol Treatment Trialists’ (CTT) Collaboration sought new individual participant data on all recorded adverse events, together with supporting information about the methods used to record and classify such events in each trial.^14^ The current analyses were restricted to trials in which the treatment was double blinded (to minimise reporting biases). We selected such trials to avoid the limitations of observational studies, notably their susceptibility to biases and confounding.^2^ We also aimed to minimise other methodological limitations (eg, the selective reporting of adverse events) that might have biased previous meta-analyses of randomised trials using only published data.^14^ The aim of the current meta-analysis was to evaluate the effects of statin therapy on muscle effects of differing severity and to explore how any excess risks varied over time, in different types of individual, and for different statin regimens.

## Methods

### Search strategy and selection criteria

Methods and analyses were prespecified.^14,15^ Briefly, all trials of statin therapy with more than 1000 participants and a scheduled mean follow-up of 2 years or more were eligible if they involved a double-blind comparison of statin versus placebo or of more intensive versus less intensive statin regimens. Statin intensity (relative reduction in LDL cholesterol from baseline) for each statin regimen was defined according to the American Heart Association and American College of Cardiology guidelines, with a low intensity classified as a less than 30% LDL cholesterol reduction, moderate intensity as a 30 to less than 50% reduction, and high intensity as a 50% or more reduction (appendix p 6).^16^ The main results were estimated separately for less intensive, moderate-intensity, and more intensive statin regimens. Analyses involved only unconfounded trials (ie, those in which there were no protocol-mandated differences between randomly assigned groups other than those created by the random allocations). We requested individual participant data on all adverse events, including the timing of such events, the timing of and reasons for stopping study treatment, non-trial statin use, use of other (non-trial) medications, comorbidities, and laboratory results (including creatine kinase for those regarding muscle; appendix p 2). Data on the exclusion criteria of potential relevance to previous intolerance to a statin are shown in the appendix (p 3). Ethics approval was granted by the UK National Health Service Health Research Authority (21/SC/0071).

### Statistical analysis

All variables for which the data were extracted were specified previously.^15^ Data were converted into a common format on the basis of the Clinical Data Interchange Standards Consortium Study Data Tabulation Model.^17^ Adverse events were mapped to a common dictionary (the Medical Dictionary for Regulatory Activities version 20.0).

The protocol prespecified that analyses of muscle symptoms would consider reported muscle pain (ie, myalgia) and weakness separately from myopathy. Before the unmasking of treatment allocation, non-myopathic muscle outcomes were categorised as: myalgia, limb pain, other musculoskeletal pain, muscle cramp or spasm, any muscle pain (ie, the combination of the previous four outcomes), muscular fatigue or weakness, and any muscle pain or weakness (ie, all the previous outcomes combined; appendix pp 4–5). We searched for creatine kinase values obtained within 2 weeks of the reported events to check for any biochemical evidence of muscle damage, and assessed the effect of the statin on the distribution of all creatine kinase values (reported as multiples of the trial-specific upper limit of normal [ULN]). Although the protocol specified that myopathy would be defined as muscle pain or weakness accompanied by creatine kinase more than ten times the ULN, it was redefined (before unmasking) as any event coded as myopathy or rhabdomyolysis owing to the frequent absence of creatine kinase data.

The log-rank observed minus expected statistic (O–E) and its variance were calculated for the first occurrence of each outcome among participants randomly assigned into each trial. The inverse-variance-weighted average of the log of the rate ratio (log RR) across all trials was then calculated as S/V (with variance 1/V, and hence with a 95% CI of S/V ±1·96/√V), where S is the sum of (O–E) and V is the sum of variance over all trials.^18,19^ Prespecified subgroup analyses involved analyses within particular participant characteristics (age, gender, race and ethnicity, history of vascular disease, history of diabetes, BMI, LDL cholesterol, and estimated glomerular filtration rate), analyses by year of treatment, and analyses of different statin regimens or intensities, or both. Tests for heterogeneity (or trend) across levels of each subgroup were performed. Further post-hoc subgroup analyses involved analyses of trials subdivided by whether they had an active run-in period, placebo run-in period, or no run-in period before random assignment and by statin solubility (ie, hydrophilic *vs* lipophilic). For exploratory analyses of the effects of statins on all events (ie, not just the first event), a negative binomial regression model (which provides additional flexibility compared with Poisson regression) was fitted in each trial to estimate the log RR and its SE. These estimates were then combined in a meta-analysis using the standard inverse-variance-weighted method.

Only two trials allowed for a direct assessment of a more intensive statin regimen versus placebo, but an indirect assessment of the effects of more intensive statin therapy was made by combining the log event RR from the 15 trials of a moderate-intensity statin regimen versus placebo (S_A_/V_A_) with the log event RR from the two trials of more intensive versus moderate-intensity statin therapy (S_B_/V_B_). Specifically, the log event RR for more intensive statin therapy versus placebo was estimated indirectly by S_A_/V_A_+S_B_/V_B_ (which had the variance 1/V_A_+1/V_B_). The overall estimate of the effect of more intensive statin therapy versus placebo was then calculated as the inverse-variance-weighted average of the direct and indirect estimates.

To estimate the average absolute effect of statin therapy on the underlying rate of specific outcomes in these trials, we applied the RR (or its lower and upper 95% CI limits) to the absolute rate in the appropriate control group. The percentage of such outcomes reported by those who were allocated statin therapy that could be attributed to such therapy was calculated as 100 × (RR–1)/RR. Note that this attributable proportion is unaffected by the absolute rate of muscle adverse event reporting in the underlying population.

We used the Wilcoxon rank-sum test to compare creatine kinase measurements recorded during follow-up between randomly assigned groups. Overall RRs were reported with 95% CIs, but all other RRs were reported with 99% CIs to provide some allowance for multiple comparisons. Analyses of all categories of muscle symptoms were done by intention-to-treat analysis and implemented using SAS version 9.4 and R version 4.1.3.

### Role of the funding source

The funder of the study had no role in study design, data collection, data analysis, data interpretation, or writing of the report

## Results

Individual participant data were available from 19 randomised double-blind trials of any statin regimen versus placebo (123 940 patients) and four randomised double-blind trials of more intensive versus less intensive statin therapy (30 724 patients; table 1). Of the 19 double-blind trials of any statin regimen versus placebo, one study^23^ compared a less intensive statin regimen versus placebo (6605 patients), 16 studies^20–22,24–32,34,35,37,38^ compared a moderate-intensity statin regimen versus placebo (95 890 patients), and two studies^33,36^ compared a more intensive statin regimen versus placebo (21445 patients).

**Figure 1:**
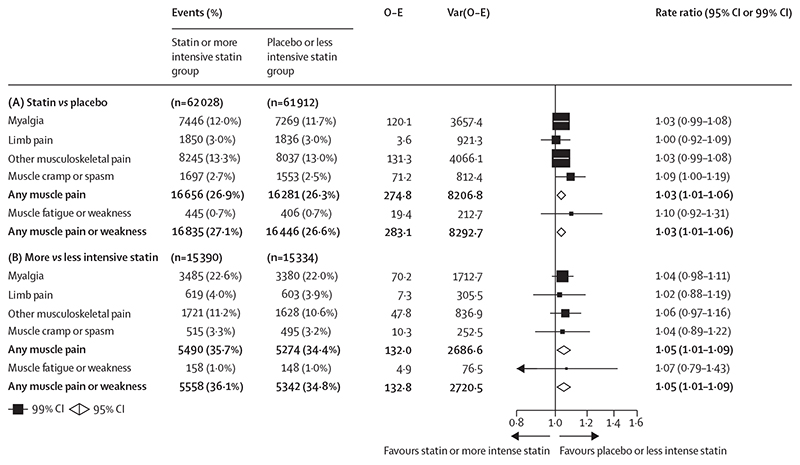
Effect on muscle adverse events in trials of any statin regimen versus placebo (A) and more versus less intensive statin regimens (B) Bold data are the totals or subtotals. O–E=observed minus expected. Var=variance.

In the 19 double-blind trials of statin versus placebo (mean age 63 years [SD 8], with 34 533 [28%] women, 59 610 [48%] participants with previous vascular disease, and 22 925 [18%] participants with diabetes), 16 835 (27·1%) participants assigned a statin versus 16 446 (26·6%) participants assigned placebo reported at least one episode of muscle pain or weakness during a median of 4·3 years. This corresponded to a 3% relative increase (RR 1·03; 95% CI 1·01–1·06; figure 1; appendix p 9). The RRs were similar (heterogeneity p=0·43) for each term used to categorise muscle symptoms (myalgia, 1·03 [0·99–1·08]; limb pain, 1·00 [0·92–1·09]; other musculoskeletal pain, 1·03 [0·99–1·08]; muscle cramp or spasm, 1·09 [1·00–1·19]; any muscle pain, 1·03 [1·01–1·06]; and muscle fatigue or weakness, 1·10 [0·92–1·31]). Figures showing trial specific findings for each category of muscle event can be downloaded from the CTT website.

**Figure 2:**
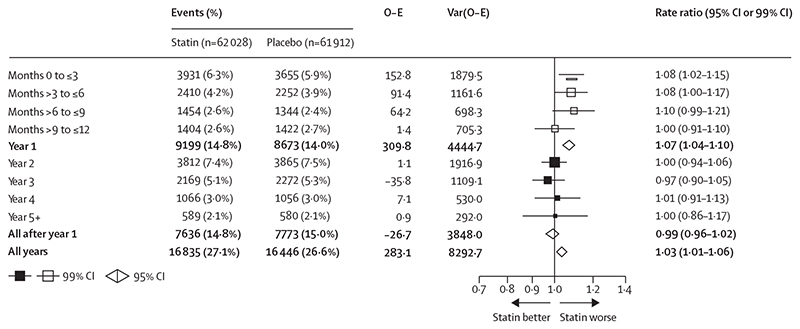
Effect on any muscle pain or weakness, by duration of treatment, in trials of any statin regimen versus placebo Bold data are the totals or subtotals. White squares indicate months, black squares indicate years. The test for heterogeneity in the log rate ratio between the first year and all subsequent years combined: χ^2^=12·1, p=0·0005. For each risk period, percentages shown are of those alive and still at risk of a first report of muscle pain or weakness at the start of the risk period. O-E=observed minus expected. Var=variance.

**Figure 3:**
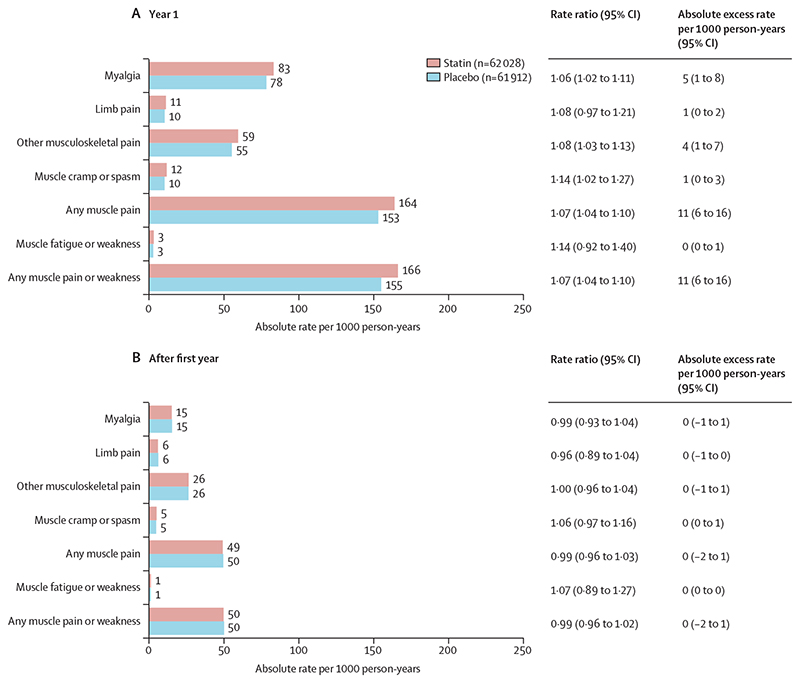
Rate ratio and absolute rate difference for muscle adverse events by duration of treatment, in trials of any statin regimen versus placebo

Statin therapy produced a 7% relative increase in muscle pain or weakness during the first year (RR 1·07; 95% CI 1·04–1·10), but no significant increase thereafter (0·99; 0·96–1·02; p value for heterogeneity for year 1 *vs* all subsequent years was 0·0005; figure 2). An increased risk was already present within the first 3 months after random assignment to treatment (1·08, 99% CI 1·02–1·15; figure 2). The aggregate rate of reporting of any muscle pain or weakness (ie, for statin and placebo groups combined) during the first year of treatment varied substantially by trial (appendix p 10), from 1·0% to 60·5%, reflecting heterogeneity in how actively information was sought. Despite this, there was no evidence that the RR for the comparison of statin versus placebo depended on the absolute reporting rate. By applying the RRs in the first and subsequent years to the average rates over these periods among patients allocated to the placebo group, allocation to a statin regimen resulted in an absolute excess rate of muscle pain or weakness of 11 (95% CI 6 to 16) per 1000 person-years in the first year and 0 per 1000 person-years (–2 to 1) in subsequent years (figure 3). Approximately 1 in 15 (calculated as [1·07–1·00]/1·07) reports of muscle symptoms (<10% of reports) during the first year were attributable to statin therapy.

When trials were subdivided by statin and statin dose, there was no evidence that the summary RRs varied significantly among different statins (heterogeneity p=0·10; appendix p 11). Nor was there any clear dose relationship for any specific statin (p value for trend all non-significant). When categorised by statin intensity (appendix p 6), less intensive and moderate-intensity regimens yielded a 3% relative increase in the rate of first reports of muscle pain or weakness (RR 1·03; 95% CI 1·00–1·05) with a 6% increase in the first year (1·06; 1·03–1·10) but no increase thereafter (0·98; 0·95–1·02; appendix p 12). There was no evidence for differences in the RR between different statins in year 1 (less intensive or moderate-intensity statin therapy, heterogeneity p=0·50; more intensive statin therapy, heterogeneity p=0·28).

**Figure 4:**
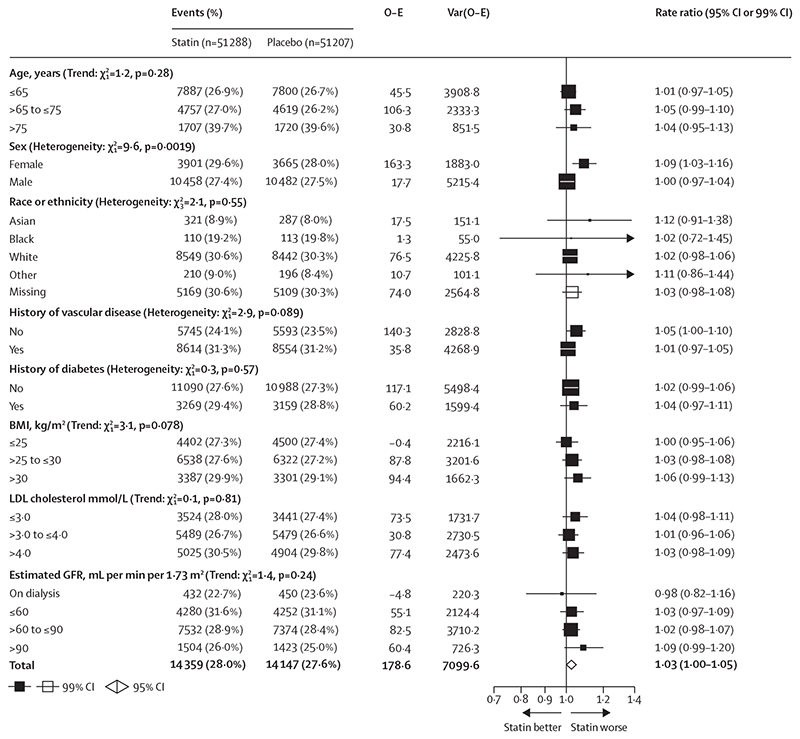
Effect of less intensive or moderate-intensity statin regimens on any muscle pain or weakness, by participant characteristics Bold indicates the overall summary result. White squares indicate missing data. Tests of heterogeneity (or trend) listed after each prognostic characteristic are of the log rate ratio for each of the subgroups of that characteristic, and are uncorrected for multiple comparisons. GFR=glomerular filtration rate. LDL=low-density lipoprotein. O–E=observed minus expected. Var=variance.

The RR in year 1 did not appear to differ according to whether there was an active or placebo pre-randomisation run-in period or no run-in, nor did it differ between whether the statin was hydrophilic or lipophilic (appendix p 13). The RR for muscle pain or weakness was greater in women for less intensive and moderate-intensity statin regimens, both during the whole follow-up period (RRs 1·09, 99% CI 1·03–1·16 in women *vs* 1·00, 0·97–1·04 in men; heterogeneity p=0·0019; figure 4) and during the first year alone when most of the excess risk was observed (so subgroup analysis should be most sensitive to variation in risk; heterogeneity p=0·012; appendix p 14). RRs did not differ significantly by other patient characteristics (figure 4; appendix p 14), or within groups of trials that exclusively recruited patients with major underlying health conditions (data not shown).

Analyses of statin intensity used data from two randomised trials of more intensive statin therapy versus placebo^33,36^ combined with data from four randomised trials of more intensive versus less intensive statin therapy that included 30 724 participants (median duration 4·9 years, mean age 62 years; SD 9 years; all with known vascular disease; table 1). As compared with a moderate-intensity regimen, more intensive statin regimens resulted in a 5% relative increase in the rate of reporting of a first episode of muscle pain or weakness (two trials RR 1·05; 95% CI 0·99–1·11; table 2; appendix pp 7,15).^39,41^ There was a similar 5% relative increase in the comparisons of different statin regimens, where both regimens fell within the moderate-intensity range (two trials 1·05; 1·00–1·11; appendix p 15).^40,42^ There were broadly similar increases in any muscle pain or weakness for any type of more-intensive versus less-intensive statin therapy, both in the first and subsequent years (heterogeneity p=0·61; appendix p 16). There was no evidence that the increase in muscle symptoms varied significantly within any of the subgroups, including sex (heterogeneity p=0·61; appendix p 17). To obtain a reliable estimate of the effects of more intensive regimens, we combined the results of a direct comparison of more intensive statin regimens versus placebo in two trials^33,36^ with the results of an indirect comparison involving two trials of more intensive versus moderate-intensity regimens^39,41^ and 16 trials of moderate-intensity regimens versus placebo.^20–22,24–32,34,35,37,38^ The direct comparison of more intensive statin regimens versus placebo during a median follow-up of 2·6 years yielded, for the rate of reporting a first episode of muscle pain or weakness, an RR of 1·09 (95% CI 1·03–1·16; table 2, appendix pp 7, 12). In the indirect comparison, the estimated RR for more intensive statin was 1·07 (1·01–1·14; table 2). Combining these direct and indirect comparisons yielded an overall RR of muscle pain or weakness with more intensive statin regimens of 1·08 (1·04–1·13), with RRs of 1·11 (1·05–1·17) in year 1 and 1·05 (0·99–1·12) after 1 year.

The RRs over the whole follow-up period for the effects of moderate-intensity or more intensive statin regimens on all reports (ie, not just the first report) of muscle pain or weakness were similar to the RRs for first events only (appendix p 7). The RRs were also similar when analyses were restricted to year 1 or to the period after year 1 (appendix p 7).

A creatine kinase concentration was available in less than 6·2% of reports of muscle pain or weakness, but for 96·7% of those cases the concentrations were less than 3 times the ULN. After excluding those with a recorded myopathy event, allocation to statin therapy resulted in a small (approximately 0·02 times the ULN) increase in median creatine kinase values during follow-up: 0·43 (IQR 0·30–0·62) times the ULN for a moderate-intensity regimen versus 0·41 (0·29–0·59) times the ULN for placebo (Wilcoxon test p<0·0001), and 0·49 (IQR 0·34–0·71) times the ULN for high intensity regimen versus 0·45 (IQR 0·31–0·65) times ULN for placebo (Wilcoxon test p<0·0001; appendix p 18). Only one trial^26^ provided information on treatment adherence in relation to muscle symptoms. In both patients allocated simvastatin and patients allocated placebo, the distribution of recorded adherence after the year 1 visit was nearly identical among those who did versus those who did not report muscle pain or weakness within the first year (appendix p 8).

Myopathy (based on Medical Dictionary for Regulatory Activities coding) was reported by 0·08% of those assigned any statin regimen versus 0·04% of those assigned placebo (RR 1·74; 95% CI 1·11–2·74, p=0·016; appendix p 19), which corresponded to an absolute excess of 0·08 (0·01–0·18) per 1000 person-years. The RR was 3·04 (1·43–6·47; p=0·0039) in year 1 and 1·28 (0·72–2·25; p=0·40) after year 1; thus, in year 1, statin therapy was the cause of approximately two-thirds ([3·04–1·00]/3·04) of myopathy cases reported by patients allocated to a statin. The RRs for less intensive and moderate-intensity (1·73 [1·05–2·87]) compared with more intensive regimens (1·78 [0·63–5·08]) were similar (appendix p 19). In analyses of more intensive versus less intensive regimens, there were few reports of myopathy with 80 mg atorvastatin once per day, but there was clear evidence of an excess with 80 mg simvastatin once per day (which is no longer approved) in the SEARCH trial^42^ compared with 20 mg simvastatin once per day (6·45; 99% CI 3·26–12·77; appendix p 20).

## Discussion

Previous randomised trials and meta-analyses of trials,^43–47^ as well as N-of-1 trials of statin therapy,^48,49^ have not been able to resolve whether statin therapy might lead to a small increased risk of muscle pain. We aimed to evaluate the causal effects of statin therapy on muscle events of differing types and severity, and to explore how any excess risk varied over time, in different types of individual, and for different statin regimens. On the basis of individual participant data on all recorded muscle events in large-scale, long-term statin trials, our study yielded many findings that enhanced understanding of the nature and risks of muscle symptoms caused by statin therapy.

Notably, we have reliably shown that the small excess risk of muscle symptoms due to statin therapy largely occurred within the first year of treatment. Other studies^12^ have shown that the risks of statin-associated muscle symptoms or statin intolerance are highest in the period after commencing therapy with a statin, but since a large proportion of such events are not due to statin therapy, such studies do not help in furthering understanding of the causal role of statins over time. There were similar excesses of risk in different types of muscle symptoms, including those categorised clinically as myalgia, muscle cramps or spasm, limb pain, other musculoskeletal pain, or muscle fatigue or weakness, but we found that muscle symptoms caused by statin therapy were no more severe than the average severity of symptoms not caused by a statin. The different statins of equivalent LDL-lowering ability included in our meta-analysis had similar effects on muscle symptoms. The relative excess was similar in different types of patients, and irrespective of how muscle symptoms were defined, hence our results are likely to be widely generalisable. Although we did not find any clear evidence of a dose-response relationship, more intensive regimens caused a greater increase in muscle symptoms than moderate-intensity regimens in the first year of treatment (11% *vs* 6%), and there was some evidence that a small excess with more intensive regimens might persist for longer than 1 year.

Based on the evidence available from the Heart Protection Study, the small excess in risk appeared to be because of events that did not usually lead to treatment discontinuation (consistent with evidence from a previous review of tabular data from statin trials),^2^ and did not result in a clinically significant change in creatine kinase. This finding indicates that most of the episodes of muscle pain or weakness caused by a statin were clinically mild. This result also indicates that, since participants with muscle symptoms generally continued treatment, the absence of any significantly increased risk of repeat reports of muscle symptoms after year 1 is not explained by patients with symptoms stopping treatment after having muscle symptoms during the first year.

We were able to explore the generalisability of our findings in several ways. Since most excess risk appears in the period immediately after treatment commences, we restricted our analyses to the first year to increase their sensitivity. There was substantial variation in the methods used to record muscle symptoms, so the first-year rates among participants allocated to the placebo group in the 19 trials of statin versus placebo also varied substantially. Nevertheless, there was little evidence that the RR for muscle pain or weakness varied according to the cumulative frequency of muscle events at 1 year. It is not clear why this is so (other than the absence of statistical power) since it might be expected that the RR should tend towards unity in those trials with higher recorded rates because of a larger proportion of misclassified events (so-called noise). However, when taken together with the general absence of variation in the relative effects of statin therapy on muscle pain or weakness in the subgroups studied, one practical conclusion is that the overall RR might be broadly generalisable in different clinical circumstances. Likewise, the absence of a sex-related difference in the trials comparing more versus less intensive regimens does not support the finding of a higher RR in women than in men in the placebo-controlled trials.

Our analyses also help to address multiple concerns that have been raised about previous analyses of the effects of statins on muscle symptoms.^50^ We found no evidence that previous estimates based on published data were seriously biased by the incomplete reporting of adverse events in trial reports. There was also no evidence that the use of an active or placebo run-in period led to the underestimation of the risk of muscle symptoms attributable to statin therapy. Finally, there was no evidence that risk estimates were biased by the exclusion of patients with comorbidities,^50^ since the relative risks were similar in the trials that had recruited exclusively from groups of patients with underlying conditions, such as those with diabetes,^30,32^ with New York Heart Association grade 2–4 heart failure,^34,35^ or patients with chronic kidney disease undergoing haemodialysis,^31,37^ with no significant heterogenity in rate ratios between trials recruiting participants with these conditions (data not shown).

In these trials, statin therapy caused approximately 11 additional reports of any muscle pain or weakness per 1000 patients during the first year, but little excess thereafter (although there was some evidence that a risk persisted beyond the first year for more intensive regimens). This finding is in contrast with the cardiovascular benefits of statin therapy, which are observed in year 1, but which are then twice as high during each subsequent year that treatment continues as previously reported (9% [99% CI 3–15%] in year 1 *vs* 24% [95% CI 21–26%] in years 1 to ≥5).^2^ Consequently, provided that statin therapy is taken for some years, the absolute reductions in major vascular events will greatly exceed any small excess of muscle symptoms that occurs soon after treatment initiation. For example, for every 1000 people in whom LDL cholesterol is lowered by approximately 1 mmol/L for 5 years (ie, an effect easily achievable in almost all patients with a moderate-intensity statin), statins might cause 11 (generally mild) episodes of muscle pain or weakness, but prevent 50 major vascular events in those with pre-existing vascular disease (secondary prevention), and 25 major vascular events in those without pre-existing vascular disease (primary prevention).^2^ Moreover, a high intensity regimen that can reduce LDL cholesterol by 50% would on average produce at least a 2 mmol/L reduction in LDL cholesterol in people with an LDL cholesterol of 4 mmol/L or more (eg, which was the measured concentration in approximately a third of people not taking a statin in UK Biobank),^51^ and would be expected to prevent twice as many major vascular events in each setting, but without any material increase in the excess rate of muscle pain or weakness as compared with a regimen reducing LDL cholesterol by 1 mmol/L. Moreover, treatment for longer than 5 years will yield even higher cardiovascular benefits.

The main strength of our analyses is that they provide the first reliable estimates of the causal contribution of statins to muscle symptoms reported by a wide range of patients. The data are derived from large-scale, double-blind, randomised trials, which guarantees the avoidance of both moderate random errors and moderate biases.^52^ By contrast, non-randomised observational studies, in which outcomes are compared between individuals who received the treatment of interest and those who did not are (irrespective of their size) prone to moderate biases (especially where participants are aware of which drugs they are taking), which cannot be guaranteed to be removed through statistical adjustment (eg, propensity score matching).^2,52^

However, there are some potential limitations to our analyses. Firstly, there was considerable heterogeneity in the methods used for the ascertainment of muscle symptoms, and definitions varied from trial to trial. Nonetheless, as noted above, the relative excesses appeared to be broadly consistent among the different trials and clinical circumstances. Secondly, although we sought adverse event data from all randomised, double-blind trials included in the CTT, some data were not available. However, the missing data represented less than 1% of all adverse events^34,36,37^ (mainly because of data privacy concerns). Furthermore, data were not consistently available on whether muscle events led to the discontinuation of allocated treatment (and thus planned on-treatment analyses for rare events were not possible), and there was no reliable information on some relevant comorbid conditions (such as hypothyroidism) or concomitant medications that might affect the risk of having symptoms. Thirdly, most reports of muscle pain or weakness were not accompanied by a measurement of creatine kinase value, so we were unable to assess whether some symptoms were associated with large increases in creatine kinase concentrations. However, among the cases for which there were data available on creatine kinase concentrations, more than 96% were less than 3 times the ULN, and there was no evidence that (after removing myopathy cases) extreme creatine kinase values were more common among participants allocated to the statin groups. Finally, although all trials excluded anyone known to have had a previous serious adverse reaction to statin therapy, and many took steps to exclude those with a previous statin sensitivity or hypersensitivity, most of them did not seek to identify or exclude participants who might now be categorised as statin intolerant (most of the trials had completed enrolment before statins were in common use and well before they were available generically).

Currently, the management of patients reporting muscle symptoms while taking statin therapy is challenging, since the belief that statin therapy often causes such symptoms is encouraged by drug labelling and other misleading sources of information (contributing to the so-called nocebo or drucebo effect, where negative expectations can lead to perceived adverse effects).^8,53^ By contrast, our results confirm that, in the majority of cases, statin therapy is not likely to be the cause of muscle pain in a person taking statin therapy. This finding is particularly true if the treatment has been well tolerated for a year or more before developing symptoms; but, even during the first year of a moderate-intensity statin regimen, it is likely to be the cause in only approximately one in 15 patients who report muscle symptoms, rising to approximately one in 10 in those who are taking a more intensive regimen. In other words, the statin is not the cause of muscle symptoms in more than 90% of individuals who report such symptoms.

In conclusion, this individual participant data meta-analysis of randomised trials has found that statin therapy causes a small proportional increase in reports of muscle pain, largely during the first year after treatment commences. These findings have shown that symptoms might differ from those observed in patients with myopathy (patients might report myalgia, cramps, limb pain, fatigue or weakness, or some other musculoskeletal pain) with no good evidence that the proportional risk increases vary between different types of patients or statins of equivalent LDL-lowering abilities, but some evidence that the proportional risk increase is higher for more intensive statin regimens than for moderate-intensity statin regimens. However, for all patients for whom statin therapy might be considered, the quantitative evidence from previous analyses of large trials within the CTT Collaboration clearly indicate that the risk is greatly outweighed by the cardiovascular benefits of statins. Our findings suggest that there is a need to review recommended strategies for managing such symptoms, and to revise the information in the drug label for statins. In particular, for patients who report mild muscle symptoms when taking a statin, our findings suggest that it is most likely that the symptoms are not due to the statin, and statin therapy should continue until other potential causes have been explored.

## Supplementary Material

Appendix

## Figures and Tables

**Table 1:** Characteristics of the trials included in the meta-analysis

	Year of publication of primary results	Number of patients	Treatment comparison, mg per day	Median follow-up, years	Baseline LDL cholesterol, mmol/L (mean [SD])	Baseline age, years (mean [SD])	Number of women (%)	Number of White participants (%)	Number of participants with a history of vascular disease (%)	Number of participants with a history of diabetes (%)	Baseline BMI, kg/m^2^(mean [SD])	Baseline estimated GFR, mL per min per 1·73 m^2^ (mean [SD])
**Statin versus placebo**											
4S^20^	1994	4444	Simvastatin (20–40 mg) *vs* placebo	5·4	4·9 (0·7)	59 (7)	827 (19%)	NA	4444 (100%)	202 (5%)	26·(3·3)	NA
WOSCOPS^21^	1995	6595	Pravastatin (40 mg) *vs* placebo	4·8	5·0 (0·5)	55 (6)	0	NA	1066 (16%)	77 (1%)	26·0(3·2)	77·8 (12·4)
CARE^22^	1996	4159	Pravastatin (40 mg) *vs* placebo	4·9	3·6 (0·4)	59 (9)	576 (14%)	3851 (93%)	4159 (100%)	586 (14%)	27·6(4·4)	67·2 (15·7)
AFCAPS/TexCAPS^23^	1998	6605	Lovastatin (20–40 mg) *vs* placebo	5·0	3·9 (0·4)	58 (7)	997 (15%)	5860 (89%)	0	155 (2%)	26·9(3·1)	65·4(11·6)
LIPID^24^	1998	9014	Pravastatin (40 mg) *vs* placebo	5·9	3·9 (0·8)	61 (8)	1516 (17%)	NA	9014 (100%)	782 (9%)	26·8(3·8)	70·6 (16·3)
LIPS^25^	2002	1677	Fluvastatin (80 mg) *vs* placebo	4·0	3·4 (0·8)	60 (10)	271 (16%)	1650 (98%)	1677 (100%)	202 (12%)	26·5(3·3)	67·6 (15·5)
HPS^26^	2002	20536	Simvastatin (40 mg) *vs* placebo	5·2	3·4 (0·8)	64 (8)	5082 (25%)	19901 (97%)	17386(85%)	5963 (29%)	27·6(4·4)	72·2 (16·5)
PROSPER^27^	2002	5804	Pravastatin (40 mg) *vs* placebo	3·3	3·8 (0·8)	75 (3)	3000 (52%)	NA	2565 (44%)	623 (11%)	26·8(4·2)	56·7(13·6)
ASCOT-LLA^28^	2003	10240	Atorvastatin (10 mg) *vs* placebo	3·3	3·4(0·7)	63 (9)	1919 (19%)	9687 (95%)	1684 (16%)	2540 (25%)	28·6 (4·6)	68·4(12·9)
ALERT^29^	2003	2102	Fluvastatin (40–80 mg) *vs* placebo	5·5	4·1(1·0)	50 (11)	715 (34%)	2039(97%)	409 (19%)	396 (19%)	25·8 (4·5)	49·6(17·0)
CARDS^30^	2004	2838	Atorvastatin (10 mg) *vs* placebo	4·2	2·9 (0·8)	61 (8)	909 (32%)	2676 (94%)	106 (4%)	2838 (100%)	28·8(3·6)	64·2(11·3)
4D^31^	2005	1255	Atorvastatin (20 mg) *vs* placebo	2·7	3·3 (0·8)	66 (8)	578 (46%)	924 (74%)	1041(83%)	1255 (100%)	27·6(4·8)	NA
ASPEN^32^	2006	2410	Atorvastatin (10 mg) *vs* placebo	4·0	2·9 (0·7)	60 (8)	811 (34%)	2029 (84%)	747 (31%)	2410 (100%)	28·9(3·8)	65·9(12·8)
SPARCL^33^	2006	4731	Atorvastatin (80 mg) *vs* placebo	4·9	3·5(0·6)	63 (11)	1908 (40%)	4415 (93%)	4731 (100%)	794 (17%)	27·9(5·2)	65·2 (13·8)
CORONA^34^	2007	4982	Rosuvastatin (10 mg) *vs* placebo	2·7	3·6 (0·9)	72 (7)	1175 (24%)	NA	4982 (100%)	1473 (30%)	26·4(3·6)	55·4(15·1)
GISSI-HF^35^	2008	4574	Rosuvastatin (10 mg) *vs* placebo	3·9	3·1 (0·9)	68 (11)	1032 (23%)	4574(100%)	4574 (100%)	1196 (26%)	27·1(4·5)	66·3 (20·4)
JUPITER^36^	2008	16714	Rosuvastatin (20 mg) *vs* placebo	1·9	2·7(0·5)	65 (8)	6374 (38%)	NA	0	44 (<1%)	27·5(3·6)	72·3 (14·8)
AURORA^37^	2009	2555	Rosuvastatin (10 mg) *vs* placebo	3·9	2·6(0·9)	64 (9)	969 (38%)	NA	1025(40%)	658 (26%)	24·8 (3·9)	NA
HOPE-3^38^	2016	12705	Rosuvastatin (10 mg) *vs* placebo	5·5	3·3 (0·9)	66 (6)	5874 (46%)	2546 (20%)	0	731 (6%)	27·1 (4·7)	79·6 (16·1)
Subtotal of the 19 statin versus placebo trials	NA	123940	NA	4·3	3·5 (0 7)	63 (8)	34533 (28%)	60152 (81%)*	59 610 (48%)	22 925 (18%)	27·2(4·1)	69·5 (15·0)
**More intensive versus less intensive statin regimen (double blind)**												
PROVE-IT^39^	2004	4162	Atorvastatin (80 mg) *vs* pravastatin (40 mg)	2·1	2·6 (0·7)^†^	58 (11)	911 (22%)	3776 (91%)	4162 (100%)	762 (18%)	29·5 (5·7)	78·8 (18·7)
A to Z^40^	2004	4497	Simvastatin (40 mg) then simvastatin (80 mg) *vs* placebo then simvastatin (20 mg)	2·0	2·1 (0·7)Ť	60 (11)	1100 (24%)	3825(85%)	4497 (100%)	1059(24%)	27·6 (4·8)	68·4(16·0)
TNT^41^	2005	10001	Atorvastatin (80 mg) *vs* atorvastatin (10 mg)	5·0	2·5 (0·5)	61 (9)	1902 (19%)	9410 (94%)	10001 (100%)	1501 (15%)	28·8 (6·1)	65·0 (12·4)
SEARCH^42^	2010	12064	Simvastatin (80 mg) *vs* simvastatin (20 mg)	7·0	2·5 (0·6)	64 (9)	2052 (17%)	11854(98%)	12064 (100%)	1267 (11%)	28·1 (4·1)	77·2(17·1)
Subtotal of the four more intensive versus less intensive trials	NA	30724	NA	4·9	2·5 (0–6)	62 (9)	5965 (19%)	28865 (94%)	30724(100%)	4589 (15%)	28·4 (5·1)	72·2 (15·6)
Total of all trials	NA	154664	NA	4.4	3·1 (0·7)	63 (8)	40 498 (26%)	89017 (85%)*	90334 (58%)	27514 (18%)	27·5 (4·3)	70·1 (15·1)

Estimated GFR was calculated using the Chronic Kidney Disease Epidemiology Collaboration equation except for CORONA and JUPITER, where the estimated GFR was supplied by trialists (and the absence of data meant that the estimated GFR could not be derived by the Cholesterol Treatment Trialists’ Collaboration); estimated GFR statistics were not presented for 4D and AURORA because these were trials done in participants who were on regular maintenance dialysis. AURORA, CORONA, and JUPITER supplied age in categorical bands, and so the midpoint of each categorical band was used as a surrogate for baseline age in all analyses. A small number of participants in the AURORA (n=218), CORONA (n=27), and JUPITER (n=1088) trials withdrew consent for use of their data post-trial, and hence data from these participants is excluded. The ASCOT-LLA trial excludes 65 patients for whom data were not available due to protocol violations. Note that some baseline characteristics might differ from previous publications because of the receipt of updated data and a broader definition of baseline vascular disease being applied in these analyses. †These two trials did not have active run-in periods; the values shown are the estimated on-treatment LDL cholesterol concentrations in the standard statin group. 4D=ie Deutsche Diabetes Dialyse Studie. 4S=Scandinavian Simvastatin Survival Study. AFCAPS/TexCAPS=Air Force/Texas Coronary Atherosclerosis Prevention Study. ALERT=Assessment of Lescol in Renal Transplantation. ASCOT-LLA=Anglo-Scandinavian Cardiac Outcomes Trial-Lipid Lowering Arm. ASPEN=Atorvastatin Study for Prevention of Coronary Heart Disease Endpoints in Non-Insulin-Dependent Diabetes Mellitus. A to Z=Aggrastat to Zocor. AURORA=A Study to Evaluate the Use of Rosuvastatin in Subjects on Regular Hemodialysis: An Assessment of Survival and Cardiovascular Events. CARDS=Collaborative Atorvastatin Diabetes Study. CARE=Cholesterol And Recurrent Events. CORONA=Controlled Rosuvastatin Multinational Trial in Heart Failure. GFR=glomerular filtration rate. GISSI-HF=Gruppo Italiano per lo Studio della Sopravvivenza nell’Insufficienza cardiaca. HOPE-3=Heart Outcomes Prevention Evaluation-3 trial. HPS=Heart Protection Study. JUPITER=Justification for the Use of Statins in Prevention: an Intervention Trial Evaluating Rosuvastatin study group. LDL=low density lipoprotein. LIPID=Long-term Intervention with Pravastatin in Ischaemic Disease. LIPS=Lescol Intervention Prevention Study. NA=not available. PROSPER=PROspective Study of Pravastatin in the Elderly at Risk. PROVE-IT=Pravastatin or Atorvastatin Evaluation and Infection Therapy. SEARCH=Study of the Effectiveness of Additional Reductions in Cholesterol and Homocysteine. SPARCL=Stroke Prevention by Aggressive Reduction in Cholesterol Levels. TNT=Treating to New Targets. WOSCOPS=West of Scotland Coronary Prevention Study. * Percentages were calculated after excluding the seven trials where information on race and ethnicity was not provided (the relevant denominators are therefore 73 832 for the 12 trials of statin *vs* placebo and 104556 for all 16 trials with information on race and ethnicity).

**Table 2:** Effects of moderate-intensity and more intensive statin regimens on any muscle pain or weakness by period of follow-up

	Events (% py)		Rate ratio (95% CI)
	Statin or more intensive statin group	Placebo or less intensive statin group	
**Direct assessments, 0–1 year**			
High-intensity statin *vs* placebo (2 trials)	1495 (15·4% py)	1351 (13·8% py)	1·11 (1·03–1·20)
Moderate-intensity statin *vs* placebo (16 trials)	7668 (18·0% py)	7282 (17·0% py)	1·07 (1·03–1·10)
High-intensity *vs* moderate-intensity (2 trials)	1259 (20·4% py)	1218 (19·6% py)	1·04 (0·96–1·12)
**Indirect assessments, 0–1 year**			
High-intensity statin *vs* placebo	NA	NA	1·10 (1·01–1·20)
**Overall (direct and indirect) assessments, 0–1 year**			
High-intensity statin *vs* placebo	NA	NA	1·11 (1·05–1·17)
**Direct assessments, after 1 year**			
High-intensity statin *vs* placebo (2 trials)	981 (6·8%py)	948 (6·4% py)	1·06 (0·97–1·16)
Moderate-intensity statin *vs* placebo (16 trials)	6616 (5·2% py)	6802 (5·4% py)	0·98 (0·95–1·02)
High-intensity *vs* moderate-intensity (2 trials)	1308 (8·5%py)	1248 (8·0% py)	1·06 (0·98–1·14)
**Indirect assessments, after 1 year**
High-intensity statin *vs* placebo	NA	NA	1·04 (0·95–1·13)
**Overall (direct and indirect) assessments, after 1 year**
High-intensity statin *vs* placebo	NA	NA	1·05 (0·99–1·12)
**Direct assessments, all years**			
High-intensity statin *vs* placebo (2 trials)	2476 (10·3% py)	2299 (9·4% py)	1·09 (1·03–1·16)
Moderate-intensity statin *vs* placebo (16 trials)	14 284 (8·4% py)	14 084 (8·3% py)	1·02 (1·00–1·05)
High-intensity *vs* moderate-intensity (2 trials)	2567 (11·9% py)	2466 (11·3% py)	1·05 (0·99–1·11)
**Indirect assessments, all years**			
High-intensity statin *vs* placebo	NA	NA	1·07 (1·01–1·14)
**Overall (direct and indirect) assessments, all years**			
High-intensity statin *vs* placebo	NA	NA	1·08 (1·04–1·13)

The table excludes one trial of a low intensity statin versus placebo (AFCAPS/TexCAPS) and two trials that compared two moderate-intensity statin regimens (SEARCH and A to Z). High intensity statin versus placebo trials: JUPITER and SPARCL. Moderate-intensity statin versus placebo trials: ALERT, ASCOT-LLA, ASPEN, AURORA, CARDS, CARE, CORONA, 4D, GISSI-HF, HOPE-3, HPS, LIPID, LIPS, PROSPER, WOSCOPS, and 4S. High intensity versus moderate-intensity, double blind trials: PROVE-IT and TNT. py=per year.

## Data Availability

Individual patient data from each contributing trial have been provided to the Cholesterol Treatment Trialists’ Collaboration on the understanding that they would be used only for the purpose of the Cholesterol Treatment Trialists’ meta-analyses and would not be released to others. Requests for such data should be made directly to the data custodians of each trial. The Cholesterol Treatment Trialists’ data policy can be found on the website: https://www.cttcollaboration.org/.

## Research in context

**Evidence before this study**

We searched Medline and the Cochrane Central Register of Controlled Trials for randomised trials, meta-analyses, or review articles published in any language between Jan 1, 1990, and June 1, 2021, that had specifically assessed the effects of statin regimens on muscle symptoms. These previous randomised trials, and several meta-analyses of published data from such trials, indicated that statins cause a small excess risk of muscle pain, but these studies might be affected by biases due to missing data. Observational studies, and meta-analyses including such studies, have also estimated the prevalence of statin-associated muscle symptoms, but such studies are subject to reporting biases and other biases, and cannot reliably establish the causal contribution of statins to such symptoms. There is a need, therefore, for a reliable assessment of the effects of various statin regimens on muscle symptoms in different clinical circumstances.

**Added value of this study**

We were able to minimise biases by restricting our analyses to large-scale, randomised, double-blind trials of statin therapy versus placebo in which there was systematic and unbiased event reporting. To overcome the potential bias arising from the selected publication of results, we obtained details of all adverse events related to muscle recorded in each individual trial participant, and coded them using the common nosological standard (from the Medical Dictionary for Regulatory Activities). The availability of individual participant data permitted more detailed analyses of risk for each statin than have previously been possible, including analyses examining the effects on particular symptoms, the timing of any excess risk, and the variation in treatment effects in different types of patients. Statin regimens caused a small relative increase (3%) in the number of first reports of muscle symptoms, but the excess of reports due to statin therapy was largely confined to the first year of treatment, during which there was an absolute excess rate of 11 (95% CI 6–16) events per 1000 person-years. Statins had similar effects on a range of reported muscle symptoms, including myalgia, muscle cramps or spasm, limb pain, and other musculoskeletal pain. There was no evidence for variation in the relative effects of different statins. The relative increase in the rate of muscle symptoms was similar in a wide range of trial participants, and was irrespective of the variation in the methods used to ascertain symptoms, suggesting that the observed relative effects of statins on muscle symptoms are likely to be generalisable. Overall, for the regimens studied in these trials, the increase in symptoms was greater for more intensive statin regimens, and a small excess risk was likely to persist for longer, than for less intensive or moderate-intensity regimens. Based on previous analyses of these trials, this excess risk of muscle symptoms is greatly outweighed by the known cardiovascular benefits of statin therapy.

**Implications of all the available evidence**

These results from randomised double-blind trials suggest that when a patient reports muscle symptoms when taking a statin, the probability that it is actually caused by the statin is low (<10%). Consequently, current recommendations on the management of such muscle symptoms should be reviewed.
