
JOURNAL INFORMATION
==============================
NLM Title Abbreviation: Lancet
Journal ID: Lancet
NLM Journal ID: 2985213R

Lancet (London, England)

ISSN: 0140-6736
EISSN: 1474-547X

ARTICLE INFORMATION
==============================
PMCID: PMC9669331
PMID: 36216002
DOI: 10.1016/S0140-6736(22)01891-8
Article ID: S0140-6736(22)01891-8
Article version: 1
Subjects: Corrections

Department of Error

Print publication date: 2022 Oct 8
Volume: 400
Issue: 10359
First page: 1194
Last page: 1194
Copyright: © 2022 The Author(s). Published by Elsevier Ltd.
Copyright year: 2022
Copyright holder: Elsevier Ltd
License: This is an open access article under the CC BY license (http://creativecommons.org/licenses/by/4.0/).
License URL: https://creativecommons.org/licenses/by/4.0/

Erratum for: Effect of statin therapy on muscle symptoms: an individual participant data meta-analysis of large-scale, randomised, double-blind trials 400 10355 10 9 2022 832 845 Lancet (London, England) 10.1016/S0140-6736(22)01545-8 PMC7613583 36049498

==============================
Cholesterol Treatment Trialists’ Collaboration. Effect of statin therapy on muscle symptoms: an individual participant data meta-analysis of large-scale, randomised, double-blind trials. Lancet 2022; 400: 832–45—In this Article, the writing committee and secretariat should have been included in the collaborator group, and collaborators Naveed Sattar's and Philip Barter's names were incorrectly spelled. In table 1, the standard deviation of the baseline LDL cholesterol for the A to Z trial should have been 0·5, and the mean baseline LDL cholesterol across all trials should have been 3·3. These corrections have been made to the online version as of Oct 6, 2022.

