# Supplementary material for: Effect of statin therapy on muscle symptoms: an individual participant data meta-analysis of large-scale, randomised, double-blind trials
Source: Lancet. Author manuscript; Available in PMC 2022 Sep 13. (PMC7613583; doi:10.1016/S0140-6736(22)01545-8)
Supplement: Supplementary file 1 [file EMS153435-supplement-Appendix.pdf]

THE LANCET

Supplementary appendix

This appendix formed part of the original submission and has been peer reviewed. We post it as supplied by the authors.

Supplement to: Cholesterol Treatment Trialists' Collaboration. Effect of statin therapy on muscle symptoms: an individual participant data meta-analysis of large-scale, randomised, double-blind trials. *Lancet* 2022; published online Aug 29. https://doi.org/10.1016/S0140-6736(22)01545-8.

Effect of statin therapy on muscle symptoms: an individual participant data meta-analysis of large-scale, randomised, double-blind trials

Online Web appendix, Table of Contents

Webtables		Page
**1**	Summary of muscle related data collected in included trials	2
**2**	Exclusion criteria of potential relevance to prior intolerance to a statin	3
**3**	Definition of muscle outcomes	4
**4**	Intensity of statin therapy	6
**5**	Effects of moderate and high intensity statin regimens on any muscle pain or weakness by duration of treatment. Analyses of both first events and of all (ie, first and subsequent) events	7
**6**	Compliance at the first visit after the first year of follow-up in MRC/BHF Heart Protection Study, by treatment allocation and occurrence of muscle pain or weakness within the first year	8

Webfigures		
**1**	Effect of statin therapy on any muscle pain or weakness, by statin intensity and trial	9
**2**	Effect of statin therapy on any muscle pain or weakness during the first year, by absolute reporting rate	10
**3**	Effect of statin therapy on any muscle pain or weakness, by statin dose and trial	11
**4**	Effect of statin therapy on any muscle pain or weakness, by statin intensity and generic name, subdivided by duration of treatment, in trials of any statin regimen vs placebo	12
**5**	Effect of low/moderate intensity statin therapy on any muscle pain or weakness during the first year, by type of run-in period and also type of statin solubility	13
**6**	Effect of low/moderate intensity statin therapy on any muscle pain or weakness during the first year, by participant characteristics	14
**7**	Effect of more vs less intensive statin therapy on any muscle pain or weakness, by trial	15
**8**	Effect of more vs less intensive statin therapy on any muscle pain or weakness, by duration of treatment	16
**9**	Effect of more vs less intensive statin therapy on any muscle pain or weakness, by participant characteristics	17
**10**	Effect of statin therapy on the distribution of creatine kinase values (reported as multiples of the upper limit of normal) during follow−up [excluding those participants who had a myopathy event]	18
**11**	Effect of statin therapy on any myopathy, by statin intensity and trial	19
**12**	Effect of more vs less intensive statin therapy on any myopathy, by trial	20

Webtable 1: Summary of muscle related data collected in included trials

Trial	Run-in type	Timing of routine FU visits	Type of event data collected	Muscle symptoms specifically enquired about at follow-up visits	CK assessed at baseline*	CK assessed at FU visits (months) *	CK assessed if muscle symptoms*
**Statin vs placebo**							
4S	Placebo	Every 6 weeks in first 18 months, then 6 monthly	All AEs	NK	Y	Every visit	NK
WOSCOPS	N (diet)	Every 3 months in first year, then 6 monthly	All AEs	N	Y	Every visit	Y
CARE	Placebo	At 6 weeks, then 3 monthly	All AEs	N	Y	Every visit	Y
AFCAPS/TexCAPS	Placebo	Every 6 weeks in first year, at 15 and 18 months, then 6 monthly	All AEs	NK	Y	Every visit	NK
LIPID	Placebo (+ diet)	Every 3 months in first year, then annually	SAEs plus some AEs	N	Y	Every 3 months	NK
LIPS	N	At 6 weeks and 6 months, then 6 monthly	All AEs	N	Y	Every visit	NK
HPS	Placebo > Active	Every 4 months in first year, then 6 monthly	SAEs plus some AEs	Y	N	None	Y
PROSPER	Placebo	Every 3 months	All AEs	N	Y	3 month visit	NK
ASCOT-LLA	N	At 6, 12 and 24 weeks, then 6 monthly	All AEs	N	N	None	N
ALERT	N	At 6 weeks then 6 monthly	All AEs	N	Y	Every visit	Y
CARDS	Placebo	At 1, 2, 3 and 6 months, then 6 monthly	All AEs	N	Y	Every visit	NK
4D	Placebo	At 1 and 6 months, then 6 monthly	All AEs	N	Y	Every visit	N
ASPEN	Placebo	At 1, 2, 3 and 6 months, then 6 monthly	All AEs	N	Y	12, 24, 36, 48	N
SPARCL	N	At 1, 3 and 6 months, then 6 monthly	All AEs	N	Y	Every visit	NK
CORONA	Placebo	At 6 and 12 weeks, then 3 monthly	All AEs	Y	Y	Every visit except 1.5	Y
GISSI-HF	N	At 1, 3 and 6 months, then 6 monthly	All AEs	Y	Y	1, 3, 6, 12, annually to 60	Y
JUPITER	Placebo	At 3 and 6 months, then 6 monthly	All AEs	N	Y	Close out visit only	NK
AURORA	N	At 3 and 6 months, then 6 monthly	All AEs	N	Y	Annually	Y
HOPE-3	Active	At 6 weeks and 6 months, then 6 monthly	SAEs plus some AEs	Y	Y	12, last visit	Y
**More intensive vs. less intensive statin (double blind)**							
PROVE-IT	N	At 2, 4 and 16 weeks, then 4 monthly	All AEs	N	Y	1, 4, 8, 16, 28	Y
A to Z	N	At 1 and 4 months, then 4 monthly	SAEs plus AEs that led to discontinuation or considered to be study-drug related	N	Y	Every visit	NK
TNT	Active	Every 3 months in first year, then 6 monthly	All AEs	N	Y	12, annually to 72	N
SEARCH	Active	At 2, 4, 8 and 12 months, then 6 monthly	SAEs plus some AEs	Y	Y	Every visit	N

* This is the planned data collection timetable, however not all data was available and/or able to be utilized.
Y=Yes; N=No; NK=not known

Webtable 2: Exclusion criteria of potential relevance to prior intolerance to a statin

Double-blind trial	Year last patient randomised*	Any statin intolerance †	Raised creatine kinase	Any statin sensitivity/ hypersensitivity‡
**Statin vs. placebo**				
4S	1989	N	N	Y
WOSCOPS	1991	N	Y (if >155 U/L)	N
CARE	1991	N	N	Y
LIPID	1992	N	N	Y
AFCAPS/TexCAPS	1993	N	N	N
HPS	1997	N	Y: CK >750 IU/L [3xULN]	N
ALERT	1997	N	N	Y
LIPS	1998	N	N	N
PROSPER	1999	N	Y: CK >3xULN	N
ASPEN	1999	N	Y: CK ≥3xULN	Y
ASCOT-LLA	2000	Y §	N (excluded those with important biochemical abnormality but CK *per se* not specified)	N
CARDS	2001	N	Y: CK ≥3xULN	Y
SPARCL	2001	N	Y: CK ≥5xULN	Y
4D	2002	N ¶	N	Y
AURORA	2004	N ¶	Y: unexplained CK >3xULN	Y
CORONA	2005	N ¶	Y: unexplained CK ≥2.5xULN	Y
GISSI-HF	2005	N	Y: CK >ULN	Y
JUPITER	2006	N ¶	Y: CK >3xULN	Y
HOPE-3	2010	N ||	Y: CK >3xULN	N
**More intensive vs. less intensive statin**				
TNT	1999	N	Y: unexplained CK >6xULN	Y
PROVE-IT	2001	Y	Y: unexplained CK >3xULN	Y
SEARCH	2001	N	Y: CK ≥3xULN	N
A to Z	2003	N **	Y: prior history of non-exercise related elevations in CK	N

Trials are ordered by the date the last patient was randomised. CK=creatine kinase; ULN=Upper limit of normal

* Most of the trials completed recruitment in the 1990s or early 2000s before statins were in common use and well before they were available generically (eg, pravastatin and simvastatin did not become available as generics until 2006). Indeed, the concept of statin intolerance/statin associated muscle symptoms only really emerged after most of the trials had been completed. Few patients in these trials would therefore have previously been exposed to statin therapy and excluded because of encountering symptoms with them. Only two of the 19 placebo-controlled trials (HPS and HOPE-3) used an active run-in and comparisons of the results in these trials with those where a placebo run-in or no run-in was used revealed similar results (Webfigure 5). Moreover, even if *some* participants from some of the trials were routinely excluded because of statin-intolerance this would not bias the estimation of *relative risks* estimated among those who *were* recruited.

† For the 2 trials where statin intolerance was used as an exclusion criterion (ASCOT-LLA and PROVE-IT), this was not specified as being muscle-related intolerance. It is therefore unlikely that significant numbers of patients from these (or the other) trials were excluded on account of what would now be classed as ‘statin intolerance’ or statin associated muscle symptoms. 6 trials (HPS, ALERT, LIPS, CORONA, HOPE-3 and SEARCH) also excluded those with chronic or inflammatory muscle disease, but these conditions are very rare and not generally thought to be statin-related.

‡ In this context statin ‘sensitivity/hypersensitivity’ would typically be taken to convey an allergic-type reaction as is conventional in trials of many classes of medication.

§ Excluded were those with any contraindications to, or previous history of, major intolerance to statins.

¶ But did exclude anyone with a known history of statin-induced myopathy.

|| But did exclude those with known ‘contraindication to statins’.

** But did exclude those with a history of non-traumatic rhabdomyolysis.

Webtable 3: Definition of muscle outcomes

Outcome	MedDRA preferred term	Lower Level Terms associated with Preferred Term
**Myalgia**	Myalgia	Generalised muscle aches; Generalized muscle aches; Localised muscle pain; Localized muscle pain; Muscle ache; Muscle burning sensation; Muscle pain; Muscle soreness; Muscle tenderness any site; Muscular pain; Muscular pains; Myalgia; Myalgia aggravated; Myalgia of lower extremities; Myalgia upper extremities; Pain muscle; Polymyalgia; Polymyalgia aggravated; Polymyalgia worsened; Tenderness muscle
	Myalgia intercostal	Intercostal myalgia; Myalgia intercostal
	Fibromyalgia	Fibromyalgia; Fibromyalgia syndrome; Fibromyalgia worsened; Fibromyositis; Fibrositis; Muscular rheumatism
**Limb pain**	Pain in extremity	Aches & pains in legs; Aching in limb; Aching pain in hands, forearm, elbows; Brachialgia; Calf pain; Foot pain; Hand pain; Leg Pain; Melalgia; Pain foot; Pain in arm; Pain in calf; Pain in extremity; Pain in fingers; Pain in foot; Pain in hand; Pain in heel; Pain in leg; Pain in limb; Pain in thigh; Pain in thumb; Pain in toe; Pain in upper extremities; Pain legs; Pain of extremities; Pain of lower extremities; Pain on lower thigh; Painful arm; Painful feet; Painful hand; Painful L arm; Painful R arm; Pains in legs; Sore feet; Unilateral leg pain
	Limb discomfort	Arm discomfort; Calf discomfort; Foot discomfort; Heaviness in extremities; Heaviness in limbs; Heavy feeling in arms & legs; Heavy feeling in arms + leg; Leg discomfort; Limb discomfort; Limb discomfort NOS; Lower extremities discomfort; Lower extremities ill feeling of; Pressure feet; Pressure in limbs; Upper extremities discomfort
	Muscle pains*	
**Other musculoskeletal pain**	Musculoskeletal pain	Arthromyalgia; Buttock pain; Interscapular pain; Musculoskeletal pain; Omalgia; Scapula pain; Shoulder blade pain; Shoulder pain
	Musculoskeletal discomfort	Back discomfort; Discomfort in joints; Groin discomfort; Hip discomfort; Muscle discomfort; Musculoskeletal discomfort; Neck discomfort; Shoulder discomfort
	Musculoskeletal chest pain	Chest tenderness; Chest tenderness of; Chest wall pain; Costal pain; Intercostal pain; Musculoskeletal chest pain; Pain lower ribs; Rib pain
	Back pain	Acute back pain; Acute lumbago; Back ache; Back distress; Back pain; Back pain (with radiation); Back pain (without radiation); Back pain aggravated; Backache; Backache, unspecified; Chronic back pain; Chronic lumbago; Dorsal pain; Dorsalgia; Loin pain; Low back ache; Low back pain; Low back pain (without radiation); Lumbago; Lumbago (excl lumbar disc lesion); Lumbalgia; Lumbar pain; Lumbar syndrome; Lumbo-sacral pain; Other back pain with radiating symptoms; Pain back; Pain dorsal; Pain loin; Pain lumbosacral; Pain over the back; Sacral pain; Sore back; Upper back pain
	Neck pain	Acute cervical pain; Cervical pain; Cervicalgia; Cervicodynia; Neck pain; Neck pain (with radiation); Pain neck
	Flank pain	Flank pain; Pain flank; Side stitch
	Groin pain	Groin pain; Inguinal pain; Pain groin

*Higher level term (HLT) – used only where preferred term or lower level term was not provided.

Webtable 3: Definition of muscle outcomes contd.

Outcome	MedDRA preferred term	Lower Level Terms associated with Preferred Term
**Muscle cramp/spasm**	Muscle spasms	Back muscle spasms; Cervical spasm; Charley horse; Cramp; Cramp in hand; Cramp legs; Cramp muscle; Cramp of limb; Cramps; Cramps calf; Cramps in legs; Cramps in the calves; Cramps leg; Cramps legs; Cramps of lower body; Cramps of lower extremities; Finger cramps; Foot cramps; Generalised spasm; Generalized spasm; Leg cramps; Muscle cramp; Muscle cramps; Muscle cramps aggravated; Muscle spasm; Muscle spasms; Muscular spasm; Neck cramps; Night cramps; Nocturnal leg muscle cramps; Spasm generalised; Spasm generalized; Spasm muscle; Spasm of muscle; Spasmophilia; Spasms; Systremma; Tetanic spasm generalised; Tetanic spasm generalized
**Muscular fatigue and weakness**	Muscular weakness	Descending muscle weakness; Generalised muscle weakness; Generalized muscle weakness; Hands weakness of; Localised muscle weakness; Localized muscle weakness; Lower extremities weakness of; Muscle weakness; Muscle weakness aggravated; Muscle weakness lower limb; Muscle weakness NOS; Muscle weakness trunk; Muscle weakness upper limb; Muscular weakness; Myasthenia; Neck muscle weakness; Neuromuscular weakness; Proximal muscle weakness; Weakness in extremity ; Weakness muscle; Weakness of arms; Weakness of limbs; Weakness of upper extremities; Weakness voluntary muscle
	Sarcopenia	Sarcopenia
	Muscle fatigue	Muscle fatigue
	Myositis	Granulomatous myositis; Interstitial myositis; Muscle inflammation; Myositis; Myotenositis; Traumatic myositis ossificans
	Muscle weakness conditions*	
**Abnormal/elevated creatine kinase**	Blood creatine phosphokinase abnormal	Blood creatine phosphokinase abnormal; Blood creatine phosphokinase abnormal NOS; Creatine phosphokinase abnormal; Plasma creatine phosphokinase abnormal; Serum creatine phosphokinase abnormal
	Blood creatine phosphokinase increased	Blood creatine phosphokinase increased; CK increased; CPK increase; CPK increased; Creatine kinase high; Creatine kinase increased; Creatine phosphokinase increased; Creatine phosphokinase serum increased; Phosphokinase creatine serum increased; Plasma creatine phosphokinase increased; Serum creatine phosphokinase increased
	Blood creatine phosphokinase MM increased	Blood creatine phosphokinase MM increased; CPK-MM increased
	Muscle enzyme increased	Muscle enzyme increased
**Myopathy (including rhabdomyolysis)**	Myopathy	Acute myopathy; Axial myopathy; Myopathy; Myopathy aggravated; Myopathy steroid; Myopathy steroid-induced; Myopathy, unspecified; Proximal myopathy; Proximal myopathy aggravated; Steroid myopathy; Symptomatic inflammatory myopathy
	Myopathy toxic	Myopathy toxic; Toxic myopathy
	Rhabdomyolysis	Muscle dissolution; Rhabdomyolysis
	Muscle necrosis	Muscle necrosis; Myonecrosis
	Necrotising myositis	Necrotising myositis; Necrotizing myositis
	Myoglobin blood increased	Blood myoglobin increased; Myoglobin blood increased
	Myoglobin blood present	Myoglobin blood present
	Myoglobin urine present	Myoglobin urine increased; Myoglobin urine present; Urine myoglobin increased
	Myoglobinaemia	Myoglobinaemia; Myoglobinemia
	Myoglobinuria	Myoglobinuria
	Myopathies*	

*Higher level term (HLT) – used only where preferred term or lower level term was not provided.

Webtable 4: Intensity of statin therapy †

Statin	Daily dosage in mg, by intensity		
	**Low (<30% LDL-C reduction)**	**Moderate (≥30% to <50% LDL-C reduction)**	**High (≥50% LDL-C reduction)**
Lovastatin	20	40 to 80	NA
Simvastatin	10	20 to 80‡	NA
Pravastatin	10 to 20	40 to 80	NA
Fluvastatin	20 to 40	2X40 or 80	NA
Atorvastatin	NA	10 to 20	40 to 80
Rosuvastatin	NA	5 to 10	20 to 40
Pitavastatin	1	2 to 4	NA

† From 2018 AHA/ACC/AACVPR/AAPA/ABC/ACPM/ADA/AGS/APhA/ASPC/NLA/PCNA Guideline on the Management of Blood Cholesterol; A Report of the American College of Cardiology/American Heart Association Task Force on Clinical Practice Guidelines; Table 3; J Am Coll Cardiol. 2019;73(24):e285-e350.

‡ Consistent with the above guideline, we classified simvastatin 80mg as 'moderate intensity' based on estimates of the median reduction in LDL-C from the VOYAGER meta-analysis (Eur Heart J Cardiovasc Pharmacother. 2016; 2: 212-7).

Webtable 5: Effects of moderate and high intensity statin regimens on any muscle pain or weakness by duration of treatment. Analyses of both first events and of all (ie, first and subsequent) events.

	First events (% py)		Rate Ratio (95% CI)	Repeated events (% py)		Rate Ratio (95% CI)
	Statin/ More statin (n=64768)	Placebo/ Less statin (n=64628)		Statin/ More statin	Placebo/ Less statin	
**0-1 year**						
**Direct assessments**						
High intensity statin vs placebo (2 trials)	1495 (15.4%)	1351 (13.8%)	1.11 (1.03 – 1.20)	1801 (18.6%)	1664 (17.0%)	1.08 (1.00 – 1.16)
Moderate intensity statin vs placebo (16 trials)	7668 (18.0%)	7282 (17.0%)	1.07 (1.03 – 1.10)	11680 (27.4%)	11102 (25.9%)	1.05 (1.02 – 1.08)
High intensity vs moderate intensity, double blind (2 trials)	1259 (20.4%)	1218 (19.6%)	1.04 (0.96 – 1.12)	1515 (24.5%)	1485 (23.9%)	1.02 (0.94 – 1.10)
**Indirect assessments**						
High intensity statin vs placebo	-	-	1.10 (1.01 – 1.20)	-	-	1.07 (0.98 – 1.17)
**Overall (direct + indirect) evidence**						
High intensity statin vs placebo	-	-	1.11 (1.05 – 1.17)	-	-	1.07 (1.02 – 1.14)
**After 1 year**						
**Direct assessments**						
High intensity statin vs placebo (2 trials)	981 (6.8%)	948 (6.4%)	1.06 (0.97 – 1.16)	1676 (11.6%)	1535 (10.4%)	1.09 (1.01 – 1.18)
Moderate intensity statin vs placebo (16 trials)	6616 (5.2%)	6802 (5.4%)	0.98 (0.95 – 1.02)	25124 (19.7%)	25102 (19.8%)	0.99 (0.97 – 1.02)
High intensity vs moderate intensity, double blind (2 trials)	1308 (8.5%)	1248 (8.0%)	1.06 (0.98 – 1.14)	2334 (15.2%)	2247 (14.4%)	1.04 (0.97 – 1.11)
**Indirect assessments**						
High intensity statin vs placebo	-	-	1.04 (0.95 – 1.13)	-	-	1.03 (0.96 – 1.11)
**Overall (direct + indirect) evidence**						
High intensity statin vs placebo	-	-	1.05 (0.99 – 1.12)	-	-	1.06 (1.00 – 1.12)
**All years**						
**Direct assessments**						
High intensity statin vs placebo (2 trials)	2476 (10.3%)	2299 (9.4%)	1.09 (1.03 – 1.16)	3477 (14.4%)	3199 (13.0%)	1.08 (1.02 – 1.15)
Moderate intensity statin vs placebo (16 trials)	14284 (8.4%)	14084 (8.3%)	1.02 (1.00 – 1.05)	36804 (21.6%)	36204 (21.3%)	1.01 (0.99 – 1.03)
High intensity vs moderate intensity, double blind (2 trials)	2567 (11.9%)	2466 (11.3%)	1.05 (0.99 – 1.11)	3849 (17.9%)	3732 (17.1%)	1.03 (0.97 – 1.08)
**Indirect assessments**						
High intensity statin vs placebo	-	-	1.07 (1.01 – 1.14)	-	-	1.04 (0.98 – 1.10)
**Overall (direct + indirect) evidence**						
High intensity statin vs placebo	-	-	1.08 (1.04 – 1.13)	-	-	1.06 (1.02 – 1.10)

Table excludes one trial of a low intensity statin vs placebo (AFCAPS/TexCAPS). High intensity statin vs placebo trials: JUPITER and SPARCL. Moderate intensity statin vs placebo trials: ALERT, ASCOT-LLA, ASPEN, AURORA, CARDS, CARE, CORONA, 4D, GISSI-HF, HOPE-3, HPS, LIPID, LIPS, PROSPER, WOSCOPS and 4S. High intensity vs moderate intensity, double blind trials: PROVE-IT and TNT

Webtable 6: Compliance at the first visit after the first year of follow-up in the MRC/BHF Heart Protection Study, by treatment allocation and occurrence of muscle pain or weakness within the first year

	Muscle pain or weakness within first year		No muscle pain or weakness within first year	
	**Simvastatin (n=2152)**	**Placebo (n=2103)**	**Simvastatin (n=8117)**	**Placebo (n=8164)**
Died within the first year	8 (<1%)	9 (<1%)	136 (2%)	159 (2%)
Withdrew within the first year	2 (<1%)	1 (<1%)	16 (<1%)	10 (<1%)
Compliance at first visit after year 1*				
<10%	247 (11%)	321 (15%)	932 (11%)	1239 (15%)
10-79%	66 (3%)	76 (4%)	168 (2%)	214 (3%)
80-89%	107 (5%)	96 (5%)	282 (3%)	300 (4%)
≥90%	1709 (79%)	1583 (75%)	6465 (80%)	6100 (75%)
Missing data	13 (1%)	17 (1%)	118 (1%)	142 (2%)

* In the majority of cases this was the 18 months visit.

Webfigure 1: Effect of statin therapy on any muscle pain or weakness, by statin intensity and trial

Trial	Dose	Events (%) Statin (n=62028)	Events (%) Placebo (n=61912)	Observed (O)−Expected (E) O−E	Observed (O)−Expected (E) Var(O−E)	Rate Ratio (95% or 99% CI)
**Low intensity**						
**Lovastatin**						
AFCAPS/TexCAPS	20−40mg	75 (2.3)	63 (1.9)	5.8	34.5	1.18 (0.76 − 1.84)
**Subtotal**		**75 (2.3)**	**63 (1.9)**	**5.8**	**34.5**	**1.18 (0.85 − 1.65)**
**Any low intensity**		**75 (2.3)**	**63 (1.9)**	**5.8**	**34.5**	**1.18 (0.85 − 1.65)**
**Moderate intensity**						
**Fluvastatin**						
ALERT	40−80mg	283 (27.0)	279 (26.5)	4.3	140.5	1.03 (0.83 − 1.28)
LIPS	80mg	47 (5.6)	51 (6.1)	−2.5	24.5	0.90 (0.54 − 1.52)
**Subtotal**		**330 (17.4)**	**330 (17.5)**	**1.7**	**164.9**	**1.01 (0.87 − 1.18)**
**Simvastatin**						
4S	20−40mg	314 (14.1)	319 (14.3)	−4.7	158.2	0.97 (0.79 − 1.19)
HPS	40mg	3564 (34.7)	3576 (34.8)	−2.0	1773.9	1.00 (0.94 − 1.06)
**Subtotal**		**3878 (31.0)**	**3895 (31.2)**	**−6.7**	**1932.1**	**1.00 (0.95 − 1.04)**
**Pravastatin**						
WOSCOPS	40mg	2069 (62.7)	2053 (62.3)	7.9	1027.6	1.01 (0.93 − 1.09)
CARE	40mg	1319 (63.4)	1305 (62.8)	3.6	655.5	1.01 (0.91 − 1.11)
LIPID	40mg	139 (3.1)	147 (3.3)	−4.8	71.5	0.94 (0.69 − 1.27)
PROSPER	40mg	1912 (66.1)	1867 (64.1)	66.1	943.2	1.07 (0.99 − 1.17)
**Subtotal**		**5439 (42.5)**	**5372 (42.0)**	**72.8**	**2697.8**	**1.03 (0.99 − 1.07)**
**Atorvastatin**						
ASCOT−LLA	10mg	944 (18.4)	934 (18.3)	4.1	469.4	1.01 (0.90 − 1.14)
CARDS	10mg	549 (38.4)	539 (38.2)	3.6	271.8	1.01 (0.87 − 1.18)
ASPEN	10mg	331 (27.3)	288 (24.0)	22.0	154.7	1.15 (0.94 − 1.42)
4D	20mg	24 (3.9)	27 (4.2)	−1.3	12.7	0.90 (0.44 − 1.86)
**Subtotal**		**1848 (22.0)**	**1788 (21.4)**	**28.4**	**908.6**	**1.03 (0.97 − 1.10)**
**Rosuvastatin**						
CORONA	10mg	321 (12.9)	299 (12.0)	10.7	155.0	1.07 (0.87 − 1.32)
GISSI−HF	10mg	1640 (71.8)	1626 (71.0)	34.2	806.4	1.04 (0.95 − 1.14)
AURORA	10mg	408 (31.9)	423 (33.2)	−3.5	207.6	0.98 (0.82 − 1.18)
HOPE−3	10mg	420 (6.6)	351 (5.5)	35.2	192.7	1.20 (1.00 − 1.45)
**Subtotal**		**2789 (22.5)**	**2699 (21.8)**	**76.5**	**1361.7**	**1.06 (1.00 − 1.12)**
**Any moderate intensity**		**14284 (29.8)**	**14084 (29.4)**	**172.8**	**7065.1**	**1.02 (1.00 − 1.05)**
**High intensity**						
**Atorvastatin**						
SPARCL	80mg	625 (26.4)	639 (27.0)	−4.2	315.9	0.99 (0.85 − 1.14)
**Subtotal**		**625 (26.4)**	**639 (27.0)**	**−4.2**	**315.9**	**0.99 (0.88 − 1.10)**
**Rosuvastatin**						
JUPITER	20mg	1851 (22.1)	1660 (19.9)	108.6	877.2	1.13 (1.04 − 1.23)
**Subtotal**		**1851 (22.1)**	**1660 (19.9)**	**108.6**	**877.2**	**1.13 (1.06 − 1.21)**
**Any high intensity**		**2476 (23.1)**	**2299 (21.5)**	**104.5**	**1193.1**	**1.09 (1.03 − 1.16)**
**All 17 low/moderate intensity trials**		**14359 (28.0)**	**14147 (27.6)**	**178.6**	**7099.6**	**1.03 (1.00 − 1.05)**
**2 high intensity trials**		**2476 (23.1)**	**2299 (21.5)**	**104.5**	**1193.1**	**1.09 (1.03 − 1.16)**
**All 19 trials**		**16835 (27.1)**	**16446 (26.6)**	**283.1**	**8292.7**	**1.03 (1.01 − 1.06)**

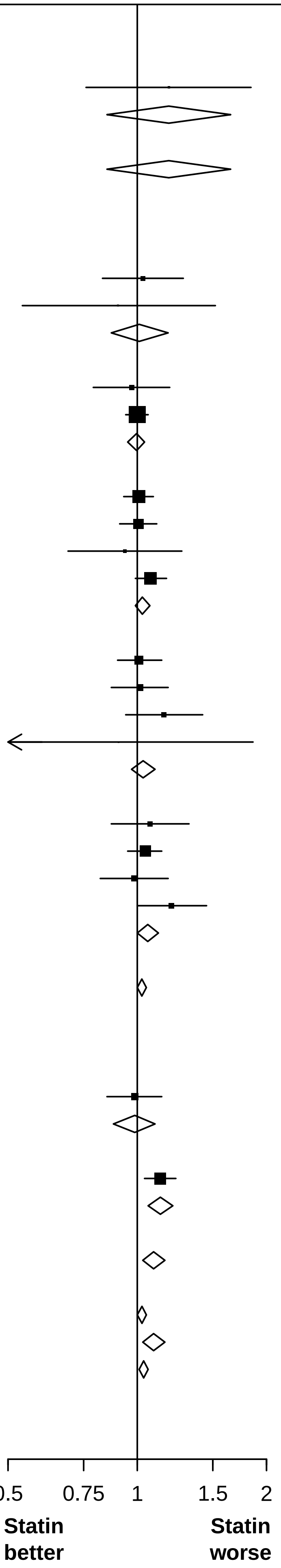

99% or 95% CI

Webfigure 2: Effect of statin therapy on any muscle pain or weakness during the first year, by absolute reporting rate

	Events (%)		Observed (O)−Expected (E)		
**Trial**	**Statin (n=62028)**	**Placebo (n=61912)**	**O−E**	**Var(O−E)**	**Rate Ratio (95% or 99% CI)**
LIPID	39 (0.9)	47 (1.0)	−4.1	21.5	0.83 (0.47 − 1.44)
AFCAPS/TexCAPS	36 (1.1)	40 (1.2)	−2.1	19.0	0.90 (0.50 − 1.62)
LIPS	16 (1.9)	15 (1.8)	0.4	7.7	1.05 (0.42 − 2.65)
4D	13 (2.1)	11 (1.7)	1.2	6.0	1.22 (0.43 − 3.49)
HOPE−3	275 (4.3)	196 (3.1)	39.9	117.7	1.40 (1.11 − 1.78)
4S	83 (3.7)	102 (4.6)	−9.5	46.2	0.81 (0.56 − 1.19)
CORONA	175 (7.0)	155 (6.2)	10.1	82.5	1.13 (0.85 − 1.50)
ASCOT−LLA	503 (9.8)	486 (9.5)	7.6	247.2	1.03 (0.88 − 1.21)
ALERT	113 (10.8)	96 (9.1)	9.4	52.2	1.20 (0.84 − 1.71)
ASPEN	148 (12.2)	123 (10.3)	12.7	67.7	1.21 (0.88 − 1.65)
SPARCL	282 (11.9)	277 (11.7)	3.5	139.7	1.03 (0.82 − 1.27)
JUPITER	1213 (14.5)	1074 (12.9)	72.4	571.4	1.14 (1.02 − 1.26)
AURORA	233 (18.2)	213 (16.7)	11.4	111.4	1.11 (0.87 − 1.41)
CARDS	276 (19.3)	248 (17.6)	13.9	130.9	1.11 (0.89 − 1.39)
HPS	2152 (21.0)	2103 (20.5)	29.4	1053.8	1.03 (0.95 − 1.11)
CARE	447 (21.5)	416 (20.0)	16.2	215.6	1.08 (0.90 − 1.28)
WOSCOPS	849 (25.7)	797 (24.2)	28.8	409.7	1.07 (0.94 − 1.22)
PROSPER	953 (33.0)	898 (30.8)	40.7	462.1	1.09 (0.97 − 1.23)
GISSI−HF	1393 (61.0)	1376 (60.1)	28.0	682.5	1.04 (0.94 − 1.15)
**All 19 trials**	**9199 (14.8)**	**8673 (14.0)**	**309.8**	**4444.7**	**1.07 (1.04 − 1.10)**

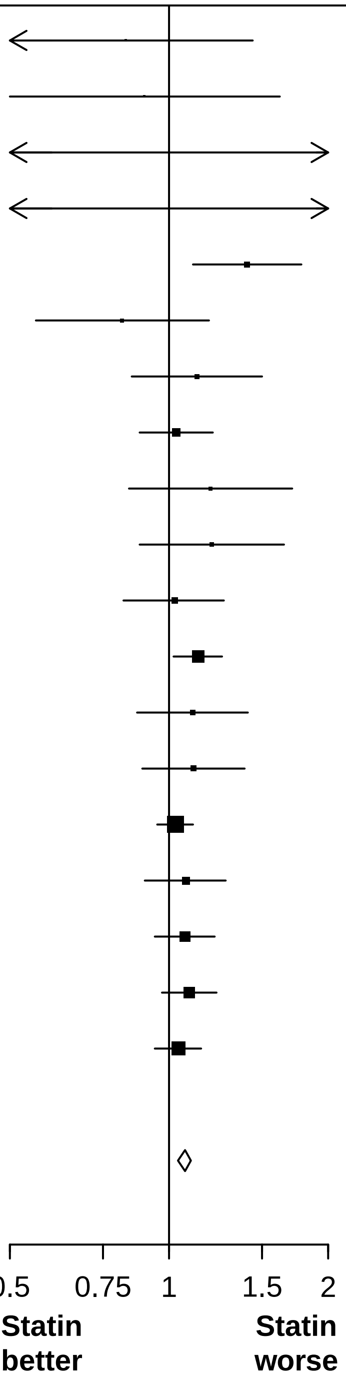

99% or 95% CI

Test for heterogeneity: χ^2_{18} =21.4, p=0.26

Test for trend: χ^2_1 =1.0, p=0.31

Webfigure 3: Effect of statin therapy on any muscle pain or weakness, by statin dose and trial

Trial	Dose	Events (%) Statin (n=62028)	Events (%) Placebo (n=61912)	Observed (O)−Expected (E) O−E	Var(O−E)	Rate Ratio (95% or 99% CI)
**Simvastatin**	Test for trend in dose:	χ^2_1 =0.1, p=0.73				
**Simvastatin 20−40mg**						
4S		314 (14.1)	319 (14.3)	−4.7	158.2	0.97 (0.79 − 1.19)
**Subtotal**		**314 (14.1)**	**319 (14.3)**	**−4.7**	**158.2**	**0.97 (0.83 − 1.13)**
**Simvastatin 40mg**						
HPS		3564 (34.7)	3576 (34.8)	−2.0	1773.9	1.00 (0.94 − 1.06)
**Subtotal**		**3564 (34.7)**	**3576 (34.8)**	**−2.0**	**1773.9**	**1.00 (0.95 − 1.05)**
**Any simvastatin**		**3878 (31.0)**	**3895 (31.2)**	**−6.7**	**1932.1**	**1.00 (0.95 − 1.04)**
**Atorvastatin**	Test for trend in dose:	χ^2_1 =0.5, p=0.46				
**Atorvastatin 10mg**						
ASCOT		944 (18.4)	934 (18.3)	4.1	469.4	1.01 (0.90 − 1.14)
CARDS		549 (38.4)	539 (38.2)	3.6	271.8	1.01 (0.87 − 1.18)
ASPEN		331 (27.3)	288 (24.0)	22.0	154.7	1.15 (0.94 − 1.42)
**Subtotal**		**1824 (23.5)**	**1761 (22.8)**	**29.7**	**895.8**	**1.03 (0.97 − 1.10)**
**Atorvastatin 20mg**						
4D		24 (3.9)	27 (4.2)	−1.3	12.7	0.90 (0.44 − 1.86)
**Subtotal**		**24 (3.9)**	**27 (4.2)**	**−1.3**	**12.7**	**0.90 (0.52 − 1.57)**
**Atorvastatin 80mg**						
SPARCL		625 (26.4)	639 (27.0)	−4.2	315.9	0.99 (0.85 − 1.14)
**Subtotal**		**625 (26.4)**	**639 (27.0)**	**−4.2**	**315.9**	**0.99 (0.88 − 1.10)**
**Any Atorvastatin**		**2473 (23.0)**	**2427 (22.6)**	**24.2**	**1224.5**	**1.02 (0.96 − 1.08)**
**Lovastatin**						
**Lovastatin 20−40mg**						
AFCAPS/TexCAPS		75 (2.3)	63 (1.9)	5.8	34.5	1.18 (0.76 − 1.84)
**Subtotal**		**75 (2.3)**	**63 (1.9)**	**5.8**	**34.5**	**1.18 (0.85 − 1.65)**
**Any Lovastatin**		**75 (2.3)**	**63 (1.9)**	**5.8**	**34.5**	**1.18 (0.85 − 1.65)**
**Fluvastatin**	Test for trend in dose:	χ^2_1 =0.4, p=0.54				
**Fluvastatin 40−80mg**						
ALERT		283 (27.0)	279 (26.5)	4.3	140.5	1.03 (0.83 − 1.28)
**Subtotal**		**283 (27.0)**	**279 (26.5)**	**4.3**	**140.5**	**1.03 (0.87 − 1.22)**
**Fluvastatin 80mg**						
LIPS		47 (5.6)	51 (6.1)	−2.5	24.5	0.90 (0.54 − 1.52)
**Subtotal**		**47 (5.6)**	**51 (6.1)**	**−2.5**	**24.5**	**0.90 (0.61 − 1.34)**
**Any Fluvastatin**		**330 (17.4)**	**330 (17.5)**	**1.7**	**164.9**	**1.01 (0.87 − 1.18)**
**Pravastatin**						
**Pravastatin 40mg**						
WOSCOPS		2069 (62.7)	2053 (62.3)	7.9	1027.6	1.01 (0.93 − 1.09)
CARE		1319 (63.4)	1305 (62.8)	3.6	655.5	1.01 (0.91 − 1.11)
LIPID		139 (3.1)	147 (3.3)	−4.8	71.5	0.94 (0.69 − 1.27)
PROSPER		1912 (66.1)	1867 (64.1)	66.1	943.2	1.07 (0.99 − 1.17)
**Any Pravastatin**		**5439 (42.5)**	**5372 (42.0)**	**72.8**	**2697.8**	**1.03 (0.99 − 1.07)**
**Rosuvastatin**	Test for trend in dose:	χ^2_1 =2.4, p=0.12				
**Rosuvastatin 10mg**						
CORONA		321 (12.9)	299 (12.0)	10.7	155.0	1.07 (0.87 − 1.32)
GISSI−HF		1640 (71.8)	1626 (71.0)	34.2	806.4	1.04 (0.95 − 1.14)
AURORA		408 (31.9)	423 (33.2)	−3.5	207.6	0.98 (0.82 − 1.18)
HOPE−3		420 (6.6)	351 (5.5)	35.2	192.7	1.20 (1.00 − 1.45)
**Subtotal**		**2789 (22.5)**	**2699 (21.8)**	**76.5**	**1361.7**	**1.06 (1.00 − 1.12)**
**Rosuvastatin 20mg**						
JUPITER		1851 (22.1)	1660 (19.9)	108.6	877.2	1.13 (1.04 − 1.23)
**Subtotal**		**1851 (22.1)**	**1660 (19.9)**	**108.6**	**877.2**	**1.13 (1.06 − 1.21)**
**Any Rosuvastatin**		**4640 (22.3)**	**4359 (21.0)**	**185.2**	**2238.9**	**1.09 (1.04 − 1.13)**
**All 19 trials**		**16835 (27.1)**	**16446 (26.6)**	**283.1**	**8292.7**	**1.03 (1.01 − 1.06)**

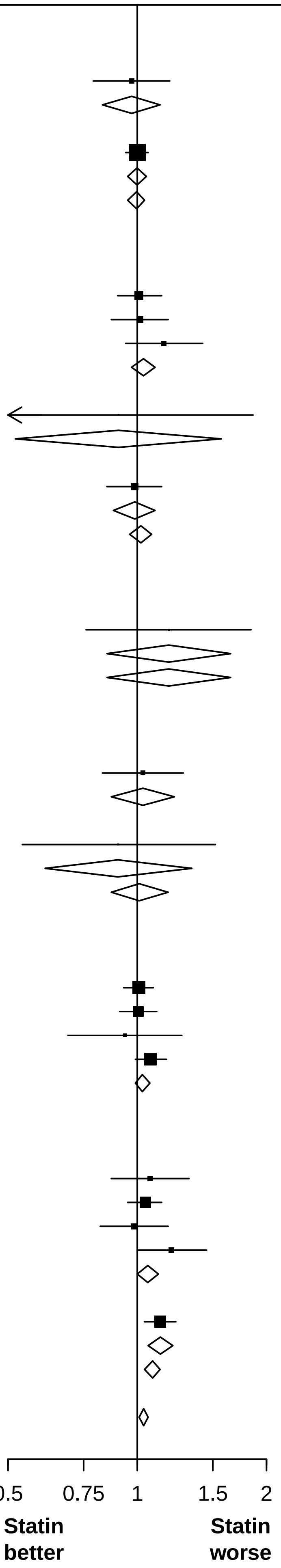

99% or 95% CI

Test for heterogeneity between 6 statin types: χ^2_5 =9.1, p=0.10

Webfigure 4: Effect of statin therapy on muscle pain or weakness, by statin intensity and generic name, subdivided by duration of treatment, in trials of any statin regimen vs placebo

	Dose	Events (%) Statin (n=62028)	Events (%) Placebo (n=61912)	Observed (O)−Expected (E) O−E	Var(O−E)	Rate Ratio (95% or 99% CI)
**YEAR 1**						
**Low/moderate intensity statin** (Heterogeneity: χ^2_5 =4.3, p=0.50)						
Simvastatin	20−40mg	2235 (17.9)	2205 (17.7)	19.9	1100.0	1.02 (0.94 − 1.10)
Pravastatin	40mg	2288 (17.9)	2158 (16.9)	81.5	1108.9	1.08 (1.00 − 1.16)
Fluvastatin	40−80mg	129 (6.8)	111 (5.9)	9.8	60.0	1.18 (0.84 − 1.64)
Lovastatin	20−40mg	36 (1.1)	40 (1.2)	−2.1	19.0	0.90 (0.50 − 1.62)
Atorvastatin	10−20mg	940 (11.2)	868 (10.4)	35.4	451.7	1.08 (0.96 − 1.22)
Rosuvastatin	10mg	2076 (16.7)	1940 (15.7)	89.4	994.0	1.09 (1.01 − 1.19)
**Any low/moderate intensity**		**7704 (15.0)**	**7322 (14.3)**	**233.9**	**3733.6**	**1.06 (1.03 − 1.10)**
**High intensity statin** (Heterogeneity: χ^2_1 =1.2, p=0.28)						
Atorvastatin	80mg	282 (11.9)	277 (11.7)	3.5	139.7	1.03 (0.82 − 1.27)
Rosuvastatin	20mg	1213 (14.5)	1074 (12.9)	72.4	571.4	1.14 (1.02 − 1.26)
**Any high intensity**		**1495 (13.9)**	**1351 (12.6)**	**75.8**	**711.1**	**1.11 (1.03 − 1.20)**
**AFTER FIRST YEAR**						
**Low/moderate intensity statin** (Heterogeneity: χ^2_5 =5.2, p=0.39)						
Simvastatin	20−40mg	1643 (16.3)	1690 (16.8)	−26.6	832.1	0.97 (0.89 − 1.06)
Pravastatin	40mg	3151 (30.5)	3214 (30.8)	−8.7	1588.9	0.99 (0.93 − 1.06)
Fluvastatin	40−80mg	201 (11.6)	219 (12.6)	−8.1	105.0	0.93 (0.72 − 1.19)
Lovastatin	20−40mg	39 (1.2)	23 (0.7)	7.9	15.5	1.66 (0.87 − 3.20)
Atorvastatin	10−20mg	908 (12.6)	920 (12.7)	−6.9	456.9	0.98 (0.87 − 1.11)
Rosuvastatin	10mg	713 (7.3)	759 (7.6)	−12.9	367.7	0.97 (0.84 − 1.10)
**Any low/moderate intensity**		**6655 (15.7)**	**6825 (16.0)**	**−55.3**	**3366.0**	**0.98 (0.95 − 1.02)**
**High intensity statin** (Heterogeneity: χ^2_1 =2.9, p=0.09)						
Atorvastatin	80mg	343 (17.0)	362 (17.8)	−7.6	176.2	0.96 (0.79 − 1.16)
Rosuvastatin	20mg	638 (9.1)	586 (8.3)	36.3	305.8	1.13 (0.97 − 1.30)
**Any high intensity**		**981 (10.9)**	**948 (10.4)**	**28.6**	**482.0**	**1.06 (0.97 − 1.16)**
**ALL YEARS**						
**Any low/moderate intensity**		**14359 (28.0)**	**14147 (27.6)**	**178.6**	**7099.6**	**1.03 (1.00 − 1.05)**
**Any high intensity**		**2476 (23.1)**	**2299 (21.5)**	**104.5**	**1193.1**	**1.09 (1.03 − 1.16)**

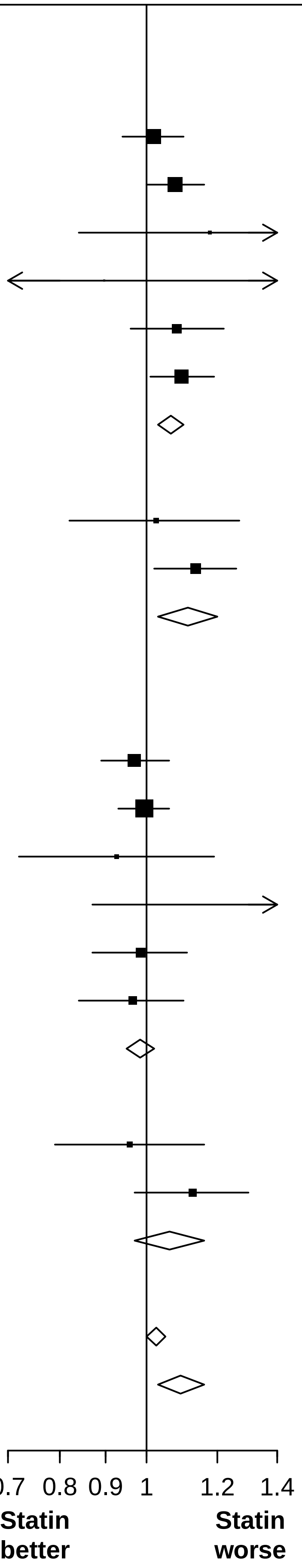

■ 99% or ◇ 95% CI

Webfigure 5: Effect of low/moderate intensity statin therapy on any muscle pain or weakness during the first year, by type of run−in period and statin solubility

Type of trial	Events (%) Statin (n=51288)	Events (%) Placebo (n=51207)	Observed (O)−Expected (E) O−E	Observed (O)−Expected (E) Var(O−E)	Rate Ratio (95% or 99% CI)
**Type of run in** (Heterogeneity: χ^2_2 =0.2, p=0.89)					
Active run−in	2427 (14.6)	2299 (13.8)	69.2	1171.4	1.06 (0.98 − 1.14)
Placebo run−in	2170 (10.5)	2040 (9.8)	79.1	1051.5	1.08 (1.00 − 1.17)
No run−in	3107 (22.4)	2983 (21.5)	85.5	1510.7	1.06 (0.99 − 1.13)
**Statin solubility** (Heterogeneity: χ^2_1 =1.7, p=0.20)					
Lipophilic	3340 (12.8)	3224 (12.4)	62.9	1630.7	1.04 (0.98 − 1.11)
Hydrophilic	4364 (17.3)	4098 (16.3)	171.0	2102.9	1.08 (1.03 − 1.15)
**All low/moderate intensity**	**7704 (15.0)**	**7322 (14.3)**	**233.9**	**3733.6**	**1.06 (1.03 − 1.10)**

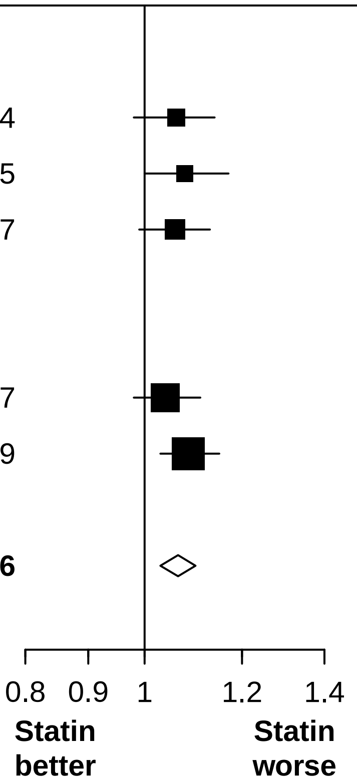

■ 99% or ◇ 95% CI

Trials with active run−in: HPS and HOPE−3.

Trials with placebo run−in: AFCAPS/TexCAPS, ASPEN, CARDS, CARE, CORONA, 4D, LIPID, PROSPER and 4S.

Trials with no run−in: ASCOT−LLA, ALERT, AURORA, GISSI−HF, LIPS and WOSCOPS.

Lipophilic statins: Atorvastatin, Simvastatin, Fluvastatin and Lovastatin.

Hydrophilic statins: Rosuvastatin and Pravastatin

Webfigure 6: Effect of low/moderate intensity statin therapy on any muscle pain or weakness during the first year, by participant characteristics

Subgroup	Events (%) Statin (n=51288)	Events (%) Placebo (n=51207)	Observed (O)−Expected (E) O−E	Observed (O)−Expected (E) Var(O−E)	Rate Ratio (95% or 99% CI)
**Age, years** (Trend: χ_1^2 =1.9, p=0.17)					
≤65	3885 (13.3)	3786 (13.0)	62.2	1907.0	1.03 (0.97 − 1.10)
>65, ≤75	2769 (15.7)	2508 (14.2)	139.5	1310.4	1.11 (1.04 − 1.19)
>75	1045 (24.3)	1023 (23.6)	33.6	512.5	1.07 (0.95 − 1.20)
**Gender** (Heterogeneity: χ_1^2 =6.3, p=0.01)					
Female	2252 (17.1)	2005 (15.3)	135.3	1057.6	1.14 (1.05 − 1.23)
Male	5452 (14.3)	5317 (13.9)	98.4	2675.6	1.04 (0.99 − 1.09)
**Race/Ethnicity** (Heterogeneity: χ_3^2 =4.6, p=0.20)					
Asian	225 (6.2)	180 (5.0)	22.7	100.8	1.25 (0.97 − 1.62)
Black	53 (9.3)	50 (8.7)	3.2	25.5	1.14 (0.68 − 1.89)
Caucasian	4982 (17.9)	4791 (17.2)	118.1	2423.2	1.05 (1.00 − 1.11)
Other	109 (4.7)	88 (3.8)	11.5	49.1	1.26 (0.87 − 1.82)
Missing	2335 (13.8)	2213 (13.1)	78.3	1134.4	1.07 (0.99 − 1.16)
**History of vascular disease** (Heterogeneity: χ_1^2 =3.6, p=0.06)					
No	2791 (11.7)	2562 (10.8)	138.2	1335.0	1.11 (1.03 − 1.19)
Yes	4913 (17.9)	4760 (17.4)	93.9	2398.0	1.04 (0.99 − 1.10)
**History of diabetes mellitus** (Heterogeneity: χ_1^2 =0.8, p=0.36)					
No	5820 (14.5)	5605 (13.9)	152.6	2838.8	1.06 (1.01 − 1.11)
Yes	1884 (16.9)	1717 (15.7)	79.6	894.1	1.09 (1.00 − 1.19)
**Body mass index, kg/m^2** (Trend: χ_1^2 =0.4, p=0.55)					
≤25	2350 (14.6)	2296 (14.0)	51.8	1154.1	1.05 (0.97 − 1.13)
>25, ≤30	3472 (14.7)	3229 (13.9)	121.8	1664.2	1.08 (1.01 − 1.15)
>30	1869 (16.5)	1786 (15.8)	63.0	907.1	1.07 (0.98 − 1.17)
**LDL−C, mmol/L** (Trend: χ_1^2 =0.1, p=0.88)					
≤3.0	2160 (17.2)	2057 (16.4)	69.7	1046.1	1.07 (0.99 − 1.16)
>3.0, ≤4.0	2939 (14.3)	2809 (13.6)	83.5	1428.0	1.06 (0.99 − 1.13)
>4.0	2387 (14.5)	2262 (13.8)	69.5	1155.8	1.06 (0.98 − 1.15)
**Estimated GFR, mL/min/1.73m^2** (Trend: χ_1^2 =0.7, p=0.42)					
On dialysis	432 (22.7)	450 (23.6)	−4.8	220.3	0.98 (0.82 − 1.16)
≤60	2417 (17.9)	2280 (16.7)	89.6	1166.8	1.08 (1.00 − 1.16)
>60, ≤90	3945 (15.1)	3773 (14.5)	91.2	1916.7	1.05 (0.99 − 1.11)
>90	849 (14.7)	777 (13.7)	45.0	402.6	1.12 (0.98 − 1.27)
**Total**	**7704 (15.0)**	**7322 (14.3)**	**233.9**	**3733.6**	**1.06 (1.03 − 1.10)**

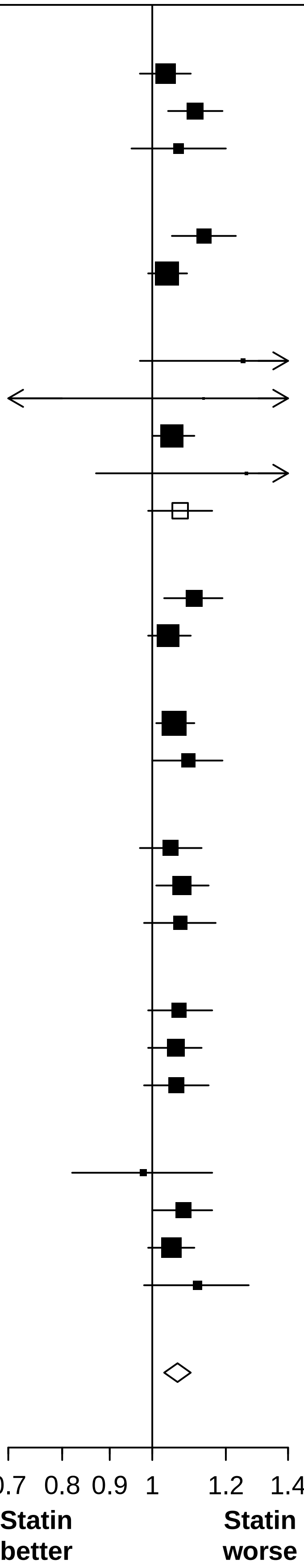

99% or 95% CI

Webfigure 7: Effect of more vs less intensive statin therapy on any muscle pain or weakness, by trial

Trial	Dose	Events (%) More Statin (n=15390)	Events (%) Less Statin (n=15334)	Observed (O)−Expected (E) O−E	Var(O−E)	Rate Ratio (95% or 99% CI)
**Comparison of moderate intensity regimens**						
A to Z	S40−80 vs S20	85 (3.8)	67 (3.0)	8.3	38.0	1.24 (0.82 − 1.89)
SEARCH	S80 vs S20	2906 (48.2)	2809 (46.6)	66.1	1425.2	1.05 (0.98 − 1.12)
**Subtotal**		**2991 (36.1)**	**2876 (34.8)**	**74.4**	**1463.2**	**1.05 (1.00 − 1.11)**
**Comparison of high vs. moderate intensity regimens**						
TNT	A80 vs A10	1944 (38.9)	1857 (37.1)	54.4	949.7	1.06 (0.97 − 1.15)
PROVE−IT	A80 vs P40	623 (29.7)	609 (29.5)	3.9	307.6	1.01 (0.87 − 1.17)
**Subtotal**		**2567 (36.2)**	**2466 (34.9)**	**58.3**	**1257.3**	**1.05 (0.99 − 1.11)**
**Total: 4 trials**		**5558 (36.1)**	**5342 (34.8)**	**132.8**	**2720.5**	**1.05 (1.01 − 1.09)**

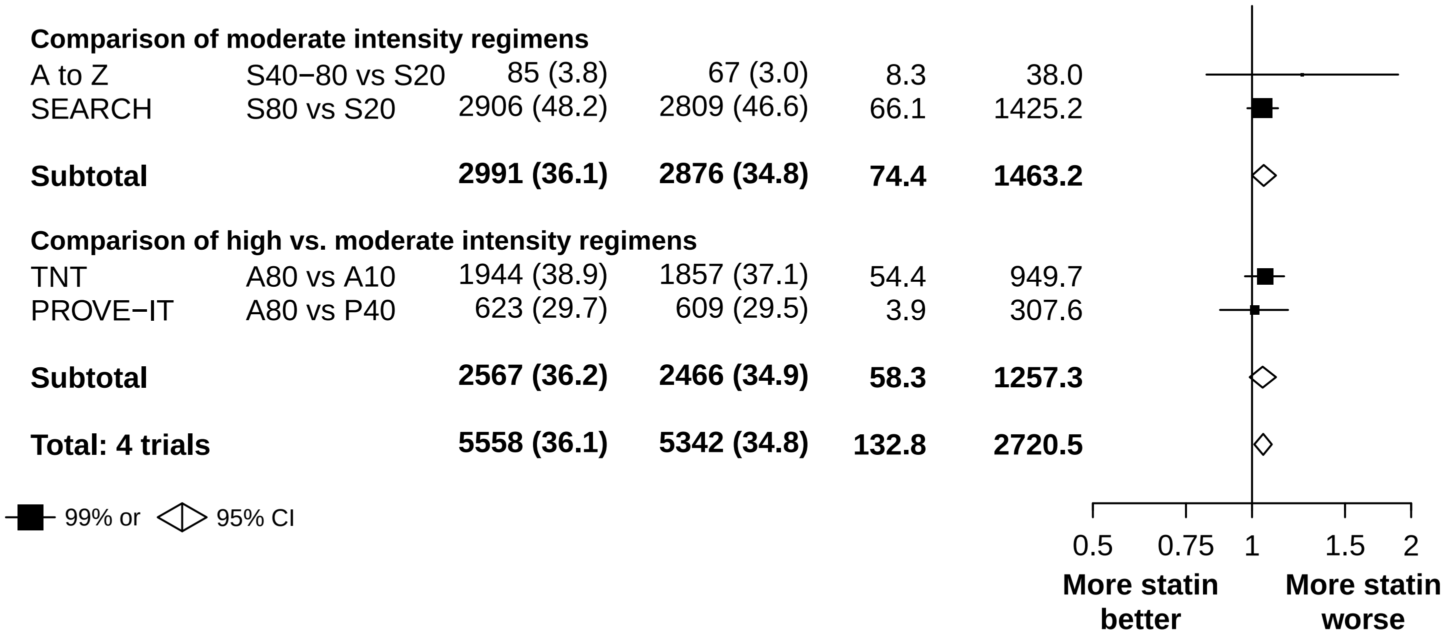

S=Simvastatin, A=Atorvastatin, P=Pravastatin.

Webfigure 8: Effect of more vs less intensive statin therapy on any muscle pain or weakness, by duration of treatment

Year	Events (%) More Statin (n=15390)	Events (%) Less Statin (n=15334)	Observed (O)−Expected (E) O−E	Observed (O)−Expected (E) Var(O−E)	Rate Ratio (95% or 99% CI)
Months 0−3	1360 (8.8)	1311 (8.5)	24.1	665.6	1.04 (0.94 − 1.15)
Months 3−6	703 (5.1)	660 (4.8)	22.8	340.0	1.07 (0.93 − 1.23)
Months 6−9	503 (3.9)	463 (3.5)	21.8	241.2	1.09 (0.93 − 1.29)
Months 9−12	390 (3.1)	417 (3.3)	−11.4	201.5	0.95 (0.79 − 1.13)
**Year 1**	**2956 (19.2)**	**2851 (18.6)**	**57.3**	**1448.3**	**1.04 (0.99 − 1.10)**
Year 2	1203 (10.1)	1197 (10.0)	7.9	599.4	1.01 (0.91 − 1.13)
Year 3	773 (8.6)	722 (8.1)	32.7	373.5	1.09 (0.96 − 1.25)
Year 4	416 (6.4)	394 (5.9)	15.9	202.4	1.08 (0.90 − 1.30)
Years 5+	210 (3.6)	178 (3.0)	18.9	97.0	1.22 (0.94 − 1.58)
**All after year 1**	**2602 (21.8)**	**2491 (20.8)**	**75.4**	**1272.3**	**1.06 (1.00 − 1.12)**
**All years**	**5558 (36.1)**	**5342 (34.8)**	**132.8**	**2720.5**	**1.05 (1.01 − 1.09)**

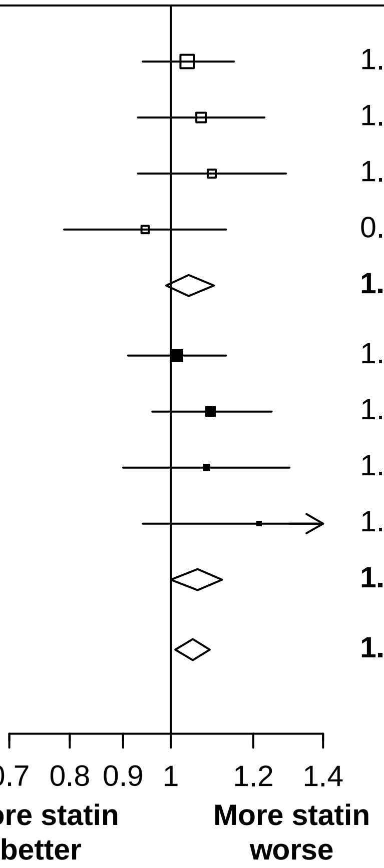

99% or 95% CI

Test for heterogeneity in the log RR between the first year and all subsequent years combined: χ^2_1 =0.3, p=0.61 .
For each risk period, percentages shown are of those alive and still at risk of a first report of muscle pain or weakness at the start of the risk period.

Webfigure 9: Effect of more vs less intensive statin therapy on any muscle pain or weakness, by participant characteristics

Subgroup	Events (%) Statin (n=15390)	Events (%) Placebo (n=15334)	Observed (O)−Expected (E) O−E	Var(O−E)	Rate Ratio (95% or 99% CI)
**Age, years** (Trend: χ^2_1 =1.1, p=0.30)					
≤65	3267 (35.0)	3202 (34.4)	49.7	1614.4	1.03 (0.97 − 1.10)
>65, ≤75	1964 (38.2)	1834 (35.9)	71.1	947.8	1.08 (0.99 − 1.17)
>75	324 (35.9)	304 (33.6)	11.3	156.5	1.08 (0.88 − 1.32)
**Gender** (Heterogeneity: χ^2_1 =0.3, p=0.61)					
Female	1161 (38.9)	1139 (38.2)	17.0	573.6	1.03 (0.93 − 1.15)
Male	4397 (35.4)	4203 (34.0)	115.7	2146.6	1.06 (1.00 − 1.12)
**Race/Ethnicity** (Heterogeneity: χ^2_3 =4.7, p=0.20)					
Asian	75 (24.8)	70 (23.9)	0.6	35.9	1.02 (0.66 − 1.56)
Black	76 (26.3)	111 (32.5)	−11.6	46.4	0.78 (0.53 − 1.14)
Caucasian	5330 (36.8)	5103 (35.5)	138.2	2604.0	1.05 (1.00 − 1.11)
Other	77 (23.5)	58 (19.1)	5.5	33.6	1.18 (0.75 − 1.84)
Missing	−	−			
**History of vascular disease**					
No	−	−			
Yes	5558 (36.1)	5342 (34.8)	132.8	2720.5	1.05 (1.00 − 1.10)
**History of diabetes mellitus** (Heterogeneity: χ^2_1 =0.2, p=0.66)					
No	4825 (36.9)	4630 (35.5)	122.7	2359.8	1.05 (1.00 − 1.11)
Yes	733 (31.9)	712 (31.1)	9.7	360.6	1.03 (0.90 − 1.18)
**Body mass index, kg/m^2** (Trend: χ^2_1 =0.1, p=0.99)					
≤25	1305 (34.4)	1263 (33.6)	10.9	640.9	1.02 (0.92 − 1.13)
>25, ≤30	2388 (37.6)	2277 (35.5)	94.5	1163.8	1.08 (1.01 − 1.17)
>30	1823 (37.0)	1770 (36.5)	23.8	896.8	1.03 (0.94 − 1.12)
**LDL−C, mmol/L** (Trend: χ^2_1 =0.3, p=0.59)					
≤2.0	1017 (31.3)	1005 (30.6)	10.0	504.5	1.02 (0.91 − 1.14)
>2.0, ≤3.0	3458 (37.4)	3315 (36.2)	94.5	1690.4	1.06 (0.99 − 1.13)
>3.0	1060 (39.5)	1003 (37.7)	27.5	514.7	1.05 (0.94 − 1.18)
**Estimated GFR, mL/min/1.73m^2** (Trend: χ^2_1 =0.7, p=0.39)					
On dialysis	−	−			
≤60	1331 (36.7)	1209 (33.6)	68.1	633.8	1.11 (1.01 − 1.23)
>60, ≤90	3214 (35.8)	3132 (35.1)	38.8	1583.9	1.02 (0.96 − 1.09)
>90	951 (40.0)	934 (39.1)	31.1	469.3	1.07 (0.95 − 1.20)
**Total**	**5558 (36.1)**	**5342 (34.8)**	**132.8**	**2720.5**	**1.05 (1.01 − 1.09)**

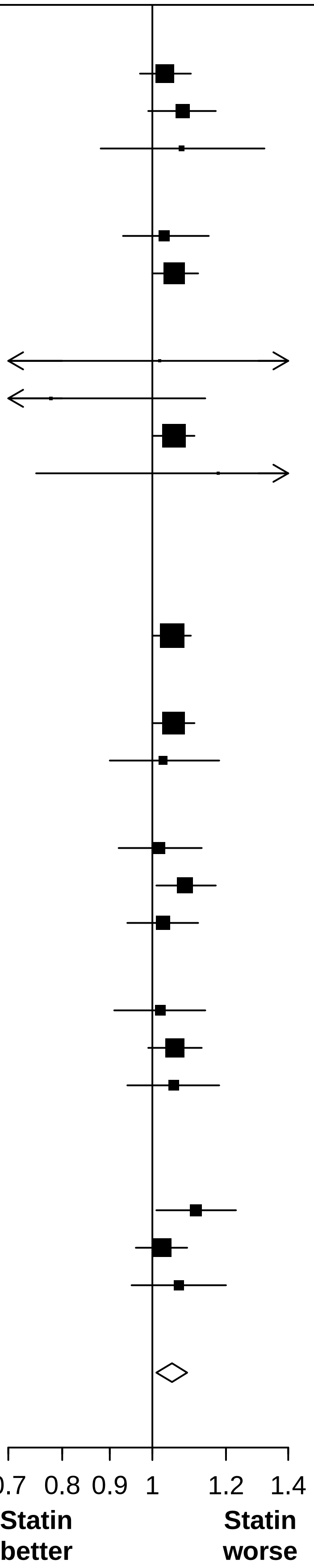

99% or 95% CI

Webfigure 10: Effect of statin therapy on the distribution of creatine kinase values (reported as multiples of the upper limit of normal) during follow−up [excluding those participants who had a myopathy event]

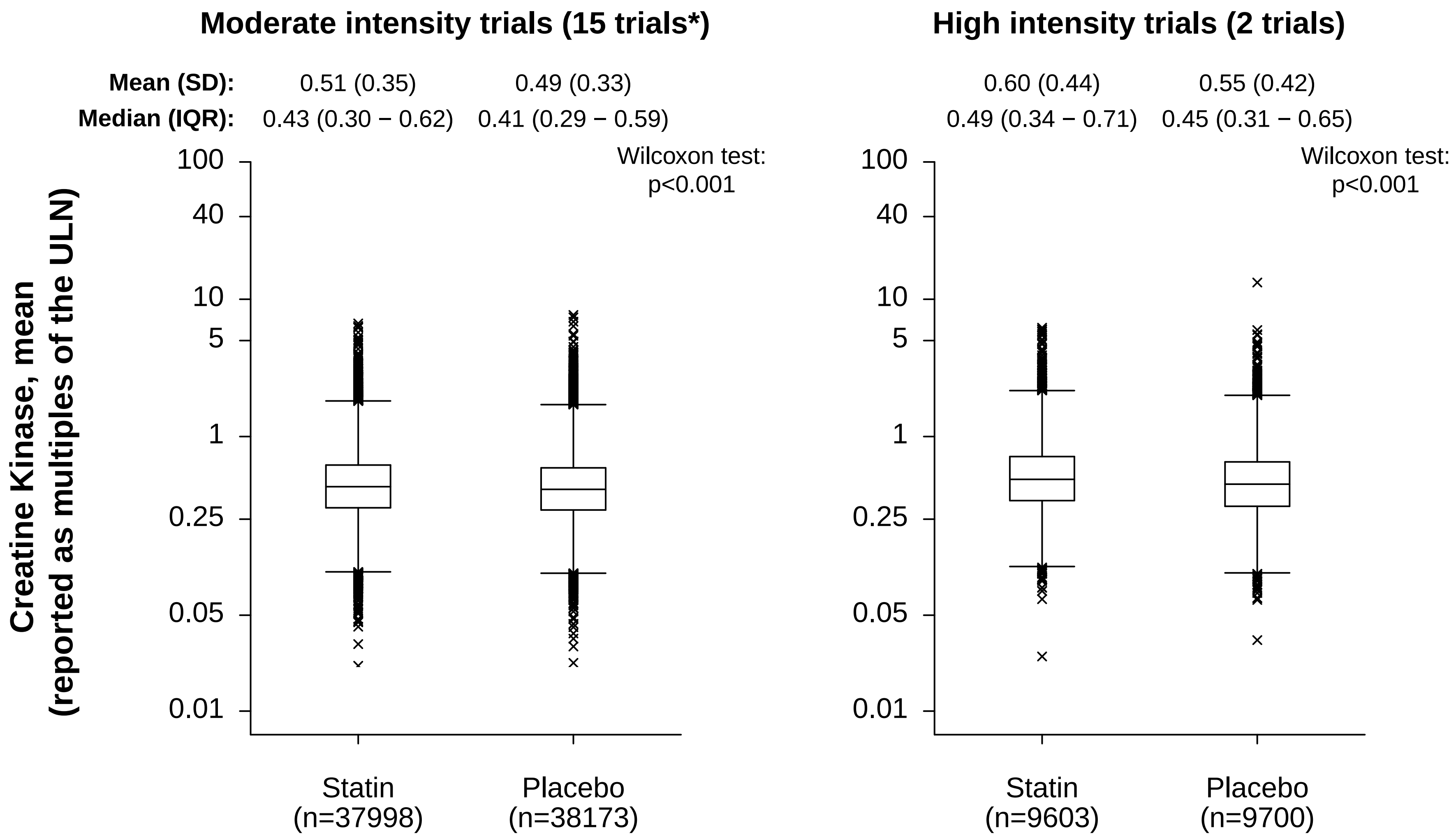

*4S did not contribute data to the above figure because it is not possible to export individual participant data from data sharing platforms.

The adverse event term of 'Abnormal or raised creatine kinase' reported 731 vs 666 events in statin and placebo arms respectively with a rate ratio (95% CI) equal to 1.09 (0.98 − 1.21).

Webfigure 11: Effect of statin therapy on any myopathy, by statin intensity and trial

Trial	Dose	Events (%) Statin (n=62028)	Events (%) Placebo (n=61912)	Observed (O)−Expected (E) O−E	Var(O−E)	Rate Ratio (95% or 99% CI)
**Low intensity**						
**Lovastatin**						
AFCAPS/TexCAPS	20−40mg	2 (<0.1)	4 (0.1)	−1.0	1.5	0.50 (0.06 − 4.13)
**Subtotal**		**2 (<0.1)**	**4 (0.1)**	**−1.0**	**1.5**	**0.50 (0.10 − 2.50)**
**Any low intensity**		**2 (<0.1)**	**4 (0.1)**	**−1.0**	**1.5**	**0.50 (0.10 − 2.50)**
**Moderate intensity**						
**Fluvastatin**						
ALERT	40−80mg	3 (0.3)	3 (0.3)	0.0	1.5	1.00 (0.12 − 8.17)
LIPS	80mg	1 (0.1)	1 (0.1)	0.0	0.5	
**Subtotal**		**4 (0.2)**	**4 (0.2)**	**0.0**	**2.0**	**0.99 (0.25 − 3.97)**
**Simvastatin**						
4S	20−40mg	1 (<0.1)	0 (0.0)	0.5	0.2	
HPS	40mg	10 (<0.1)	4 (<0.1)	3.0	3.5	2.34 (0.59 − 9.26)
**Subtotal**		**11 (<0.1)**	**4 (<0.1)**	**3.5**	**3.7**	**2.52 (0.92 − 6.93)**
**Pravastatin**						
WOSCOPS	40mg	0 (0.0)	0 (0.0)	0.0	0.0	
CARE	40mg	2 (<0.1)	0 (0.0)	1.0	0.5	
LIPID	40mg	5 (0.1)	2 (<0.1)	1.5	1.7	2.34 (0.33 − 16.41)
PROSPER	40mg	1 (<0.1)	1 (<0.1)	0.0	0.5	
**Subtotal**		**8 (<0.1)**	**3 (<0.1)**	**2.5**	**2.7**	**2.47 (0.76 − 8.07)**
**Atorvastatin**						
ASCOT−LLA	10mg	1 (<0.1)	0 (0.0)	0.5	0.2	
CARDS	10mg	1 (<0.1)	0 (0.0)	0.5	0.2	
ASPEN	10mg	0 (0.0)	1 (<0.1)	−0.5	0.2	
4D	20mg	3 (0.5)	2 (0.3)	0.5	1.2	1.52 (0.15 − 15.20)
**Subtotal**		**5 (<0.1)**	**3 (<0.1)**	**1.0**	**2.0**	**1.64 (0.41 − 6.55)**
**Rosuvastatin**						
CORONA	10mg	3 (0.1)	2 (<0.1)	0.5	1.2	1.48 (0.15 − 14.84)
GISSI−HF	10mg	2 (<0.1)	0 (0.0)	1.0	0.5	
AURORA	10mg	2 (0.2)	1 (<0.1)	0.5	0.7	1.96 (0.10 − 38.45)
HOPE−3	10mg	2 (<0.1)	1 (<0.1)	0.5	0.7	1.94 (0.10 − 37.98)
**Subtotal**		**9 (<0.1)**	**4 (<0.1)**	**2.5**	**3.2**	**2.16 (0.73 − 6.40)**
**Any moderate intensity**		**37 (<0.1)**	**18 (<0.1)**	**9.4**	**13.7**	**1.99 (1.17 − 3.37)**
**High intensity**						
**Atorvastatin**						
SPARCL	80mg	6 (0.3)	5 (0.2)	0.5	2.7	1.20 (0.25 − 5.69)
**Subtotal**		**6 (0.3)**	**5 (0.2)**	**0.5**	**2.7**	**1.20 (0.37 − 3.93)**
**Rosuvastatin**						
JUPITER	20mg	3 (<0.1)	0 (0.0)	1.5	0.7	7.52 (0.38 − 147.33)
**Subtotal**		**3 (<0.1)**	**0 (0.0)**	**1.5**	**0.7**	**7.52 (0.78 − 72.33)**
**Any high intensity**		**9 (<0.1)**	**5 (<0.1)**	**2.0**	**3.5**	**1.78 (0.63 − 5.08)**
**All 17 low/moderate intensity trials**		**39 (<0.1)**	**22 (<0.1)**	**8.4**	**15.2**	**1.73 (1.05 − 2.87)**
**2 high intensity trials**		**9 (<0.1)**	**5 (<0.1)**	**2.0**	**3.5**	**1.78 (0.63 − 5.08)**
**All 19 trials**		**48 (<0.1)**	**27 (<0.1)**	**10.4**	**18.7**	**1.74 (1.11 − 2.74)**

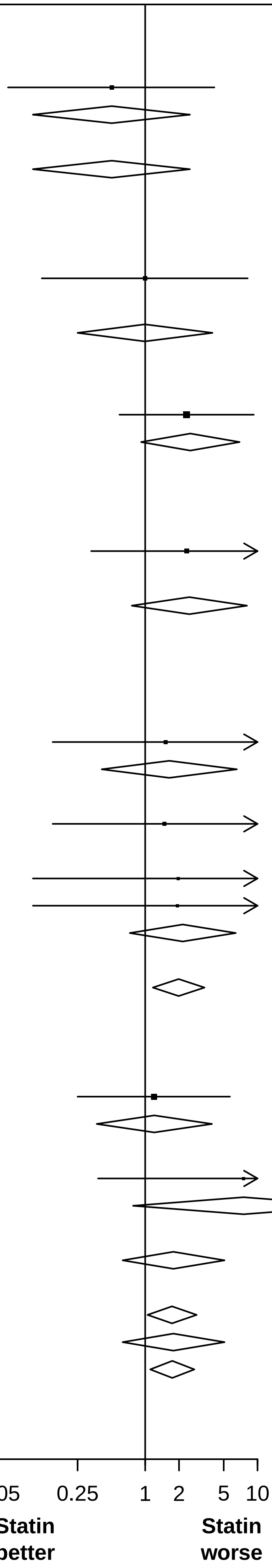

99% or 95% CI

Webfigure 12: Effect of more vs less intensive statin therapy on any myopathy, by trial

Trial	Dose	Events (%) More Statin (n=15390)	Events (%) Less Statin (n=15334)	Observed (O)−Expected (E) O−E	Observed (O)−Expected (E) Var(O−E)	Rate Ratio (95% or 99% CI)
**Comparison of moderate intensity regimens**						
A to Z	S40−80 vs S20	4 (0.2)	1 (<0.1)	1.5	1.2	3.26 (0.33 − 32.68)
SEARCH	S80 vs S20	55 (0.9)	2 (<0.1)	26.6	14.2	6.45 (3.26 − 12.77)
**Subtotal**		**59 (0.7)**	**3 (<0.1)**	**28.0**	**15.5**	**6.11 (3.71 − 10.05)**
**Comparison of high vs. moderate intensity regimens**						
TNT	A80 vs A10	11 (0.2)	11 (0.2)	0.0	5.5	1.00 (0.33 − 3.00)
PROVE−IT	A80 vs P40	2 (<0.1)	0 (0.0)	1.0	0.5	
**Subtotal**		**13 (0.2)**	**11 (0.2)**	**1.0**	**6.0**	**1.18 (0.53 − 2.62)**

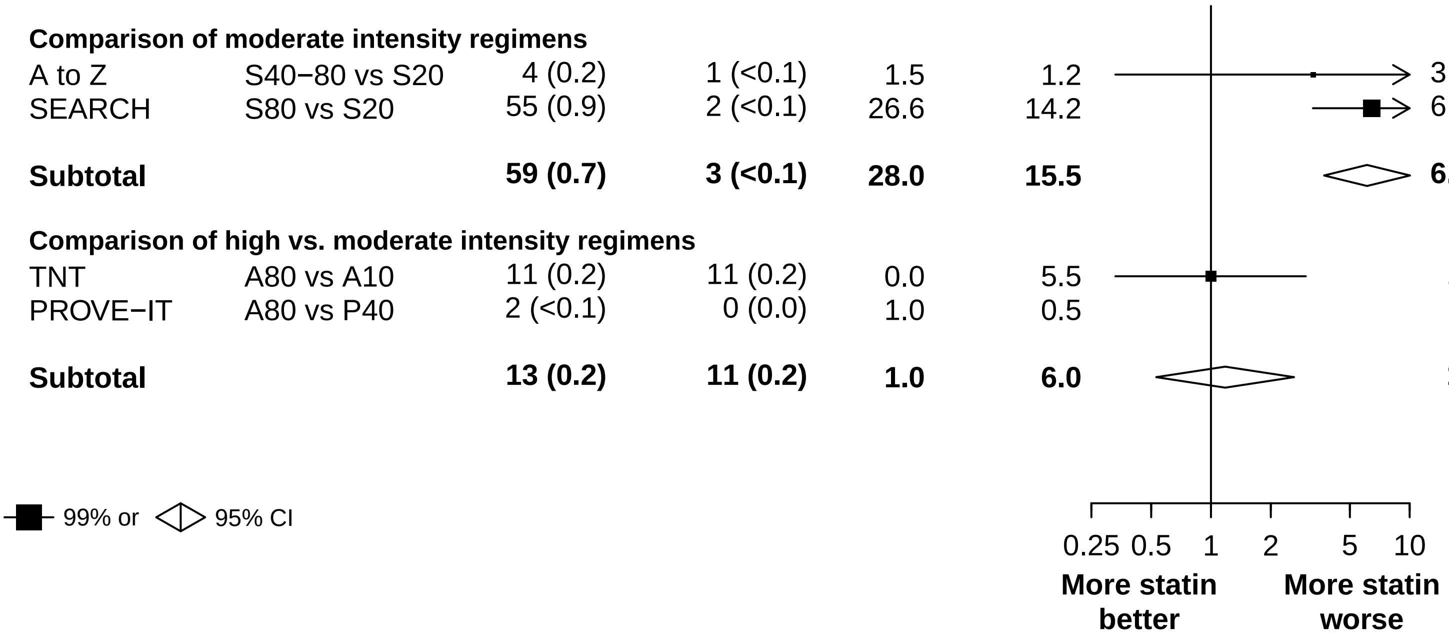

S=Simvastatin, A=Atorvastatin, P=Pravastatin.
